# PIK3CA mutations-mediated downregulation of circLHFPL2 inhibits colorectal cancer progression via upregulating PTEN

**DOI:** 10.1186/s12943-022-01531-x

**Published:** 2022-05-26

**Authors:** Xiaodan Chong, Jingde Chen, Nanxin Zheng, Zhuqing Zhou, Yanan Hai, Shiqing Chen, Yu Zhang, Qingzhuo Yu, Shijun Yu, Zhiqin Chen, Wenfang Bao, Ming Quan, Zhe-Sheng Chen, Yangyang Zhan, Yong Gao

**Affiliations:** 1 Clinical Oncology Institute, Translational Medicine Center, Navy Military Medical University, 800 Xiangyin Road, Yangpu District, Shanghai, 200433 China; 2 Department of Oncology, Shanghai East Hospital, Tongji University School of Medicine, 150 Jimo Road, Pudong District, Shanghai, 200120 China; 3 Department of Colorectal Surgery, Changhai Hospital, Navy Military Medical University, 168 Changhai Road, Yangpu District, Shanghai, 200433 China; 4 Department of Gastrointestinal Surgery, Shanghai East Hospital, Tongji University School of Medicine, 150 Jimo Road, Pudong District, Shanghai, 200120 China; 5 The Medical Department, 3D Medicines Inc., Shanghai, 201114 China; 6 Department of Pharmaceutical Sciences, College of Pharmacy and Health Sciences, St. John’s University, Queens, NY 11439 USA; 7 Department of Pharmacy, Shanghai Eastern Hepatobiliary Surgery Hospital, Navy Military Medical University, 225 Changhai Road, Yangpu District, Shanghai, 200433 China

**Keywords:** Colorectal cancer, PI3KCA mutation, circLHFPL2, miR-556-5p, miR-1322, PTEN

## Abstract

**Background:**

PIK3CA mutation and PTEN suppression lead to tumorigenesis and drug resistance in colorectal cancer (CRC). There is no research on the role of circular RNAs (circRNAs) in regulating PIK3CA mutation and MEK inhibitor resistance in CRC.

**Methods:**

The expression of circLHFPL2 in PIK3CA-mutant and wild-type cells and tissues was quantified by RNA-sequencing and qRT-PCR. CCK-8 assay and colony formation assay were used to evaluate cell viability. Annexin V/PI staining was implemented to assess cell apoptosis. Luciferase assay, biotin-coupled microRNA capture, and RIP assay were used to validate the interaction among potential targets. Western blotting and qRT-PCR assays were used to evaluate the expression of involved targets. Xenograft tumor in a nude mouse model was used to explore the role of circRNAs in vivo.

**Results:**

RNA sequencing defined downregulated expression of circLHFPL2 in both PIK3CA^H1047R^ (HCT116) and PIK3CA^E545K^ (DLD1) cells. CircLHFPL2 was also downregulated in PIK3CA-mutant CRC primary cells and tissues, which was correlated with poor prognosis. CircLHFPL2 was mainly localized in the cytoplasm and its downregulation was attributed to the PI3K/AKT signaling pathway activated by phosphorylating Foxo3a. CircLHFPL2 inhibited PI3KCA-Mut CRC progression both in vitro and in vivo. Furthermore, our work indicated that circLHFPL2 acts as a ceRNA to sponge miR-556-5p and miR-1322 in CRC cells and in turn modulate the expression of PTEN. Importantly, circLHFPL2 was able to overcome PIK3CA-mediated MEK inhibitor resistance in CRC cells.

**Conclusions:**

Downregulation of circLHFPL2 sustains the activation of the PI3K/AKT signaling pathway via a positive feedback loop in PIK3CA-mutant CRC. In addition, downregulation of circLHFPL2 leads to MEK inhibitor resistance in CRC. Therefore, targeting circLHFPL2 could be an effective approach for the treatment of CRC patients harboring oncogenic PIK3CA mutations.

**Supplementary Information:**

The online version contains supplementary material available at 10.1186/s12943-022-01531-x.

## Introduction

Class I phosphoinositide 3-kinase (PI3K) is a dimeric enzyme which phosphorylates phosphatidylinositol 4,5 bisphosphate (PIP2) to phosphatidylinositol 3,4,5-trisphosphate (PIP3) [1]. PIP3 plays a pivotal role as a second cellular messenger to activate downstream AKT signaling pathway and modulates various biological processes including cell proliferation, cell cycle, and motility [2]. PI3K contains a catalytic and a regulatory subunit [3]. The catalytic subunit consists of four isoforms including p110a, p110β, p110γ and p110δ. PI3KCA encodes p110α and is one of the most frequently mutated oncogenes in colorectal cancer (CRC) [4–6]. Mutations of PIK3CA (Phosphatidylinositol-4, 5-bisphosphate 3-kinase catalytic subunit alpha) lead to a sustained activation of the PI3K/AKT signaling pathway in a growth factor-independent manner, promoting the growth and invasion of CRC cells [6, 7].

Most PIK3CA mutations commonly occur in two regions: an acidic cluster (E545K) in the helical domain and a histidine residue (H1047R) in the kinase domain of p110α protein [8]. E545K and H1047R are the most frequently observed p110 α somatic mutations in human cancers that induce downstream AKT activation in the absence of growth factor stimulation [9]. In cells without PIK3CA mutation, receptor tyrosine kinases (RTKs) are activated by growth factors, and p110α is brought to the cell membrane through the binding of p85 and phospho-IRS1, therefore converting PIP2 to PIP3. In cancer cells harboring a p110α helical domain mutation, the p110a mutant directly binds to IRS1, thereby being recruited to the cell membrane and converting PIP2 to PIP3 without RTKs activation [7]. Dysregulation of the PI3K signaling pathway is commonly associated with tumorigenesis and drug resistance such as resistance to MEK inhibitors, while the underlying mechanism needs to be further investigated [10–12].

Circular RNAs (circRNAs) are a subset of endogenous non-coding RNAs (ncRNAs), consisting of a closed loop structure connecting the 5′ and 3′ ends [13]. CircRNAs are essential for diverse regulatory mechanisms, including competing endogenous RNAs (ceRNAs), transcriptional regulation, protein interactions and translational regulation [14–16]. In CRC, circRNAs act as sponges of miRNAs to abrogate the inhibition of miRNAs on their target genes, thereby regulating diverse biological processes, including cell growth, apoptosis, migration and invasion [17–19]. However, whether circRNAs are involved in PI3K downstream activation and drug resistance of CRC remains largely unknown.

In the present study, we reported that circLFHPL2 downregulated by PIK3CA mutation mediates FOXO3a phosphorylation and inhibits PTEN expression by sponging miR-553 and miR-1266 in CRC. We further demonstrated that circLHFPL2 is involved in PIK3CA mutation-mediated MEK inhibitor resistance by downregulating P-gp and BCRP. Targeting circLHFPL2 could be an effective approach for the treatment of CRC patients harboring tumor mutations of this gene.

## Materials and methods

### Patients and samples

We collected tumor tissues from CRC patients diagnosed between January 2014 and December 2015 in Changhai Hospital with the following inclusion criteria: 1) patients were diagnosed with CRC by using biopsy pathology; 2) pathological result was identified as adenocarcinoma, or mucinous adenocarcinoma; 3) CRC was the first and only malignant tumor without metastasis; 3) neoadjuvant therapy was not administered before surgery; 4) radical resection was performed and tumor tissue samples were obtained, snap-frozen in liquid nitrogen, and stored at − 80 °C at the time of surgery; 5) sufficient clinicopathological information and successful follow-up data were collected. Preoperative characteristics consisted of age, gender, primary tumor location, body mass index (BMI), preoperative serum CEA and CA199. Postoperative pathological characteristics included tumor size, histology, T stage, N stage, tumor deposit, intravascular invasion, and perineural invasion, and were evaluated according to the seventh AJCC/TNM staging system. The mutation statuses on PIK3CA Exon-9 (E545K or E542K) and Exon-20 (H1047R or H1047L) were routinely tested.

Finally, a total of 1124 CRC cases were included in our study. Among them, 36 (3.20%) CRC cases had a PIK3CA mutation. To adjust for significant clinicopathologic covariates between PIK3CA mutation and WT groups, a propensity score matching (PSM) was implemented to reduce the possibility of selection bias by using a logistic regression model. All the variables at baseline were used as criteria to calculate the propensity scores. PSM produced 30 cases in PIK3CA mutation and 30 cases in WT groups. All the clinicopathological characteristics in these two groups showed no statistical difference (*P* ≥ 0.05; Table 1). Then, the tumor tissues from these 60 CRC cases were used for qRT-PCR to detect the expression of circLHFPL2, miR-556-5p and miR-1322, and for Western blotting or IHC staining to detect the protein levels of PTEN, P-glycoprotein (P-gp/ABCB1) and Breast cancer resistance protein (BCRP/ABCG2).

**Table 1:** Comparison of baseline characteristics of CRC cases with WT or Mutant PIK3CA status before and after propensity score matching (PSM)

Characteristic	Before PSM			After PSM		*P* value
	WT cohort (*n*=1088)	Mutant cohort (*n*=36)	*P* value	WT cohort (*n*=30)	Mutant cohort (*n*=30)	
Age^a^ (years)	62.0 (54.0-69.0)	65.0 (51.0-73.2)	0.055	59.6±14.0	63.4±13.2	0.2893
Sex			0.081			0.7961
Male	685	17		13	15	
Female	403	19		17	15	
Tumor location			<0.001			0.607
Rectum	577	9		6	9	
Left colon	288	7		5	6	
Right colon	223	20		19	15	
BMI (kg/m^2^)			0.015			0.289
<18.5	43	5		1	4	
18.5-23.9	588	17		19	14	
>23.9	457	14		10	12	
CEA level (U/ml)			0.323			1.000
≥5	375	11		8	7	
<5	674	22		19	20	
Unknown	39	3		3	3	
CA199 level (U/ml)			<0.001			0.510
≥37	168	16		7	11	
<37	882	17		20	16	
Unknown	38	3		3	3	
Tumor size^a^ (cm)	4.0 (3.0-5.5)	4.8 (3.0-6.0)	0.421	4.8 (4.0-6.0)	4.5 (3.1-6.0)	0.572
Histology			<0.001			0.785
Well/moderately differentiated Adenocarcinoma	901	20		21	19	
Poorly differentiated Adenocarcinoma/Mucinous adenocarcinoma	187	16		9	11	
T stage			0.306			0.998
T_1_	50	1		0	1	
T_2_	219	2		0	2	
T_3_	695	26		24	22	
T_4_	124	7		6	5	
Nstage			0.016			0.135
N_0_	633	12		15	12	
N_1_	312	16		7	14	
N_2_	143	8		8	4	
Tumor deposit			0.004			1.000
Positive	150	12		6	6	
Negative	928	24		24	24	
Unknown	10	0		0	0	
Intravascular invasion			0.001			1.000
Positive	82	9		5	4	
Negative	996	27		25	26	
Unknown	10	0		0	0	
Perineural invasion			0.055			1.000
Positive	109	8		5	4	
Negative	969	28		25	26	
Unknown	3	0		0	0	

^a^Except these, other values were summarized as frequencies and percentages

### Cell lines and cell cultures

CRC cell lines including HCT116, DLD1, HT29, SW480, LoVo, and RKO cells were purchased from the American Type Culture Collection (ATCC, Washington, D.C., USA) and these cell lines were tested negative for mycoplasma. HCT116 cells with PIK3CA^WT^ and PIK3CA^H1047R^, and DLD1 cells with PIK3CA^WT^ and PIK3CA^E545K^ were established as previously described [6]. All cells were cultivated with McCoy’s 5A medium (Thermo, Waltham, USA) supplemented with 10% fetal bovine serum (FBS, Invitrogen; Thermo Fisher Scientific, Inc.), penicillin and streptomycin (100 U/ml; Invitrogen; Thermo Fisher Scientific, Inc.) in a humidified incubator (Thermo, Waltham, USA) with 5% CO_2_ at 37 °C. For MEK inhibitor treatment, AZD6244 or RDEA199 was dissolved in DMSO (concentration set to 0, 0.01, 0.1, 1, 10 μM) and added into cell culture medium for 24 h.

### CircRNA sequencing

Three batches of RNA samples were extracted from each cell lines (HCT116 cells with PIK3CA WT or E545K mutation and DLD1 cells with PIK3CA WT or H1047R mutation) and quantified by NanoDrop ND-1000 (Thermo, Waltham, USA). The RNA samples were then subjected to library construction and sequenced on Illumina Hiseq 4000 according to the guidelines of the Illimina TruSeqTM Stranded Total RNA Library Prep Kit [20]. RNA sequencing was conducted by Majorbio (Shanghai, China). Differential expression of circRNAs was analyzed by the limma package of R software. Significant differentially expressed circRNAs were screened by |Log_2_fold change| > 1and *p* value < 0.05.

### Cell transfection and establishment of circLHFPL2 overexpressing cells

CRC cells were seeded into 6-well plates. After cultivation to 70% confluence, miRNA mimics, small interfering RNAs (siRNAs) and corresponding controls were transfected into cells using Lipofectamine™ 3000 (L3000008; Invitrogen; Thermo, Waltham, USA) according to the manufacturer’s instructions. The siRNA sequences are shown in Additional file 1. The plasmids containing an empty vector or full-length circLHFPL2 were subcloned into pCDH-CMV-MCS-EF1-PURO lentivirus vector to establish the stable cell lines. Puromycin was used to select clone cells successfully infected with the virus, and qRT-PCR was used to confirm the expression of circLHFPL2 in the cells.

### RNA extraction and qRT-PCR

Total RNA was extracted using TRIzol reagent (Invitrogen, Thermo, Waltham, USA) following the manufacturer’s guidelines [21]. cDNA was obtained through reverse transcription of total RNA with the PrimeScript RT Master Mix (Takara Bio, Inc., Dalian, China) from 300 ng of RNA. The abundance of circRNA was determined by qPCR with SYBR Premix Ex Taq II kit (Takara Bio, Inc., Dalian, China) according to the manufacturer’s instructions. GAPDH was used to normalize the expression of circLHFPL2 and PTEN (Supplementary Fig. 4), and U6 was used to normalize the expression of miR-556-5p and miR-1322. The relative abundance of expression was calculated with the 2^-ΔΔCt^ method. The sequences of all the primers are shown in Additional file 1: Table S1.

### Western blotting assay

Total protein was isolated with RIPA buffer (Beyotime Institute of Biotechnology, Shanghai, China) supplemented with EDTA-free protease inhibitor cocktail (cat. no. 04693159001; Roche Diagnostics GmbH). The BCA assay kit (Thermo, Waltham, USA) was used to determine the protein concentrations. Then, proteins were separated on 10% SDS-PAGE and transferred to PVDF membranes. The membranes were then blocked in 5% nonfat milk for 2 h at room temperature. Primary antibodies were used to incubate the membranes at dilution ratios recommended by the manufacturers at 4 °C overnight. Then, horseradish peroxidase-conjugated secondary antibodies with a dilution ratio of 1:5000 were used to incubate the membranes at room temperature for 1 h. Finally, enhanced chemiluminescence (Tanon Science and Technology, Shanghai, China) was used to develop the blots and the reaction bands were visualized in an imaging system (Tanon 4600, Tanon Science and Technology, Shanghai, China). GAPDH was used as an internal control. All the information of antibodies used in our study was shown in Additional file 2: Table S2.

### Cell viability assay

Cell Count Kit-8 (CCK8, Takara Bio, Inc., Dalian, China) assay was used to assess the viability of CRC cells as described previously [22]. Briefly, 1.5 × 10^3^ cells per well were seeded into 96-well plates and maintained in medium containing 0.5% FBS. After being cultured for 1d, 2d, 3d, 4d and 5d, 10 μL CCK-8 solution was added into each well of the 96-well plates for 1.5 h at 37 °C. The absorbance was measured at 450 nm with spectrophotometer (BioTek Instruments, Inc., Winooski, VT, USA) and then calculated as described before.

### Colony formation assay

The treated CRC cells were digested and seeded into 6-well plates at a density of 1 × 10^3^ cells/well. Then, cells were cultured in medium containing 0.5% FBS and maintained in an incubator with 5% CO_2_ at 37 °C for 14 d. Subsequently, cells were washed with PBS and fixed with paraformaldehyde for 10 min at room temperature. The fixed cells were stained with 0.1% crystal violet (Sigma-Aldrich; Merck KGaA, Darmstadt, Germany) for 20 min.

### Cell apoptosis assay

The treated cells were cultured in medium containing 0.5% FBS and maintained in an incubator with 5% CO_2_ at 37 °C for 48 h. Then, cells were digested and washed twice with PBS and resuspended in buffer. Next, Annexin V-fluorescein isothiocyanate apoptosis detection kit (Becton, Dickinson and Company, New Jersey, USA) was utilized according to the manufacturer’s instructions. The cell apoptosis analysis was conducted with flow cytometry using the BD FACSVantage™ SE System (Becton, Dickinson and Company, New Jersey, USA).

### Luciferase reporter assay

The pmirGLO luciferase reporter plasmids (Promega, Madison, WI, USA) of circLHFPL2-WT and circLHFPL2-Mut were designed with or without a 3′-untranslated region binding site for miR-556-5p and miR-1322. PTEN-WT and PTEN-Mut pmirGLO luciferase reporter plasmids were designed using the same approach. pmirGLO luciferase reporter plasmids were co-transfected into the cells with miRNA mimics or NC mimics using Lipofectamine 3000 Reagent (Invitrogen, Waltham, USA). Then, the Dual-Luciferase Reporter Assay Kit (Promega, Madison, USA) was used to measure the activities of Luciferase and Renilla according to the manufacturer’s instructions.

### Biotin-coupled miRNA capture

The 3′ end of biotinylated miR-556-5p and miR-1322 mimics or control RNA (Ribio, Guangzhou, China) were transfected into 1 × 10^6^ HCT116 and DLD1 cells at a final concentration of 50 nM for 48 h before harvest. Then the cell pellet was incubated with 0.7 mL lysis buffer (5 mM MgCl_2_, 100 mM KCl, 20 mM Tris [pH 7.5], 0.3% NP-40, 50 U of RNase OUT (Invitrogen, Waltham, USA) and complete protease inhibitor cocktail (Roche Applied Science, Basel, Switzerland) on ice for 10 min. The biotin-coupled RNA complex was pulled down by incubating the cell lysates with streptavidin-coated magnetic beads (Thermo, Waltham, USA) and centrifugation at 10,000×g for 10 min. The abundance of circLHFPL2 in the bound fraction was evaluated by qRT-PCR analysis.

### In vivo experiment

The protocols of animal experiments were reviewed and approved by the Animal Care Committee of Changhai Hospital. Six-week-old C57BL/6 nude mice were purchased from Model Animal Research Center of Nanjing University (Nanjing, China) and maintained according to the guidelines of the National Institutes of Health. Treated cells (3 × 10^6^, 100 μL) were subcutaneously injected in the left flank of the mice. Tumor size was determined every 3 days as previously reported (Length x Width^2^ × 0.5). After 30 days, the mice were sacrificed, and the tumor weight was measured. For MEK inhibitor treatment, 10 mg/Kg of AZD6244 was injected intraperitoneally every 3 days into tumor models. Tumor size was determined every 3 days as previously reported (Length x Width^2^ × 0.5).

### Statistical analysis

Kaplan-Meier method was used to perform survival analysis and a log-rank test was used for comparison. Student’s t test was performed to compare the statistical difference of two groups and one-way ANOVA analysis was conducted for multiple groups. Pearson’s correlation analysis was performed between the expression levels of two groups of RNAs. Data are plotted as mean ± SEM. A significantly statistical difference was determined at *P* < 0.05.

## Results

### CircLHFPL2 is downregulated in PIK3CA-mutant CRC cells and tissues

**Fig. 1:**
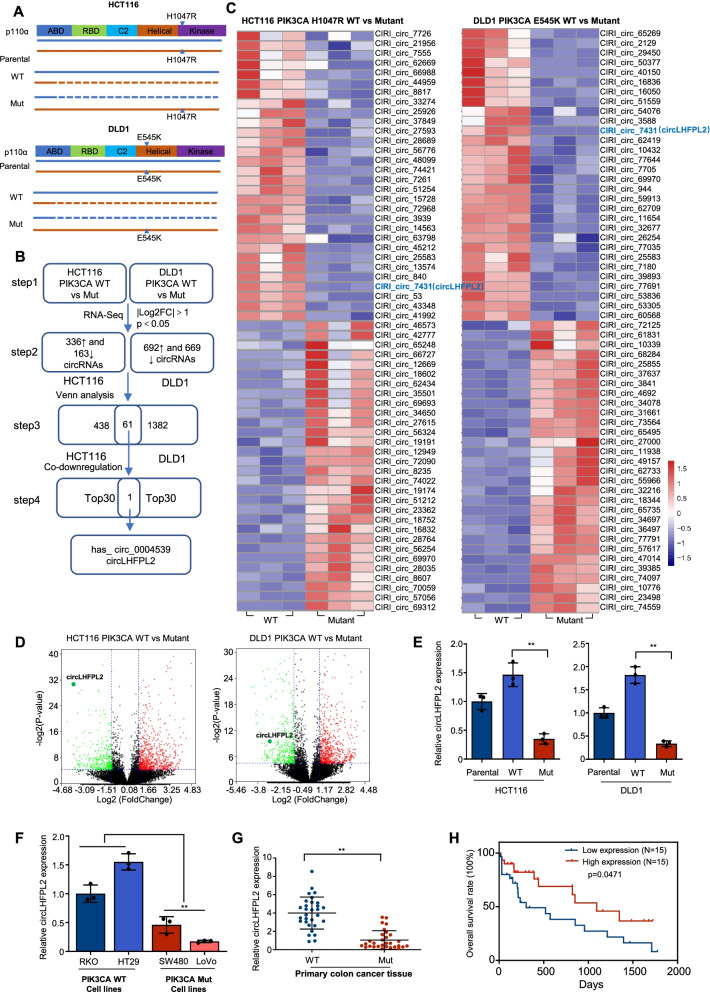
CircLHFPL2 is downregulated in PIK3CA-mutant CRC cells and tissues. **A** PIK3CA parental, WT and mutant allele configurations of CRC lines. **B** The flowchart delineates the steps for identifying and validating circRNAs in PIK3CA-mutant CRC cells. **C** Hierarchical clustering showing the top 30 upregulated and downregulated circRNAs in HCT116 PIK3CA^H1047R^ (left) and DLD1 PIK3CA^E545K^ (right) cells. **D** Volcano plots showing differential expression between the two different conditions. **E** Relative expression of circLHFPL2 determined via qRT-PCR in parental, WT and Mut HCT116 (left) and DLD1 (right) cells. **F** Relative expression of circLHFPL2 determined via qRT-PCR in CRC cell lines. **G** Relative expression of circLHFPL2 determined via qRT-PCR in tumor tissues of 30 CRC patients with PIK3CA Mut and 30 patients with PIK3CA WT. **H** Survival analysis in patients with different expression pattern of circLHFPL2 with Kaplan-Meier method. Data are presented as mean ± SEM; *n* ≥ 3. *p < 0.05; **p < 0.01

Most PIK3CA mutations are clustered in two common regions, with H1047R in the kinase domain and E545K in the helical domain. It was found that the CRC cell line HCT116 harbors a heterozygous H1047R mutation, whereas DLD1 CRC cells have a heterozygous E545K mutation (Fig. 1A). We evaluated these cell lines in situations where either the WT or the mutant allele of PIK3CA was knocked out (Fig. 1A). The cells in which the mutant allele was disrupted and the WT allele was intact was named ‘WT’ (Fig. 1A), whereas the cells where only the WT allele was disrupted and the mutant allele was intact was named ‘mutant’ (Mut, Fig. 1A). To define the expression profiles of circRNA in PIK3CA-mutant CRC cells, circRNA sequencing by Hiseq, which captured 78,346 circRNAs, was implemented using HCT116 cells with PIK3CA^WT^ or PIK3CA^H1047R^ as well as DLD1 cells with PIK3CA^WT^ or PIK3CA^E545K^ (step1 as shown in Fig. 1B). In HCT116 cells with WT or Mut PIK3CA, 336 circRNAs were upregulated and 163 circRNAs were downregulated, while in DLD1 cells with WT or Mut PIK3CA, 692 circRNAs were upregulated and 669 circRNAs were downregulated (step2 as shown in Fig. 1B). Thirty of the most downregulated or upregulated circRNAs in HCT116 and DLD1 cells with PIK3CA mutation are shown in the heatmap (step 4 as shown in Fig. 1A, B and C). CircLHFPL2 was the only circRNA found among the top dysregulated circRNAs in both cell lines (step 4 as shown in Fig. 1B, C and D). Then, we validated the expression of circLHFPL2 in the parental, WT and Mut HCT116 cells and DLD1 cells. The results confirmed that the expression of circLHFPL2 was significantly downregulated in HCT116 cells with PIK3CA^H1047R^ compared with that in the parental or HCT116 PIK3CA^WT^ cells (Fig. 1E). Similarly, the expression of circLHFPL2 was also remarkably downregulated in DLD1 cells with PIK3CA^E545K^ compared with that in the parental DLD1 cells or those with PIK3CA^WT^ (Fig. 1F). Then, we investigated the expression profiles of circLHFPL2 in other CRC cell lines. Figure 1E showed that circLHFPL2 was significantly downregulated in PIK3CA-mutant cell lines including SW480 (PIK3CA^H1047R^ cells) and LoVo (PIK3CA^P449T^ cells) but not in PIK3CA WT cell lines (RKO and HT29). Furthermore, we collected tumor tissues from 30 CRC patients with PIK3CA mutations and 30 patients without PIK3CA mutations, and analyzed the expression pattern of circLHFPL2 in these tissues. The patients had no significant difference in TNM stage and tumor size (Table 1). We found that expression of circLHFPL2 was significantly lower in PIK3CA-mutant CRC tissues than that of WT PIK3CA tissues (Fig. 1G). We also observed that LHFPL2 mRNA was downregulated in PIK3CA-mutant CRC tissues (Supplementary Fig. 5A). Moreover, survival analysis revealed that in PIK3CA-mutant patients, those with relatively low expression of circLHFPL2 was significantly associated with poor overall survival (Fig. 1H). These results demonstrate that circLHFPL2 is downregulated in PIK3CA-mutant CRC cell lines and tissues, and downregulation of circLHFPL2 is correlated with poor prognosis.

### CircLHFPL2 is the target gene of the transcription factor Foxo3a

**Fig. 2:**
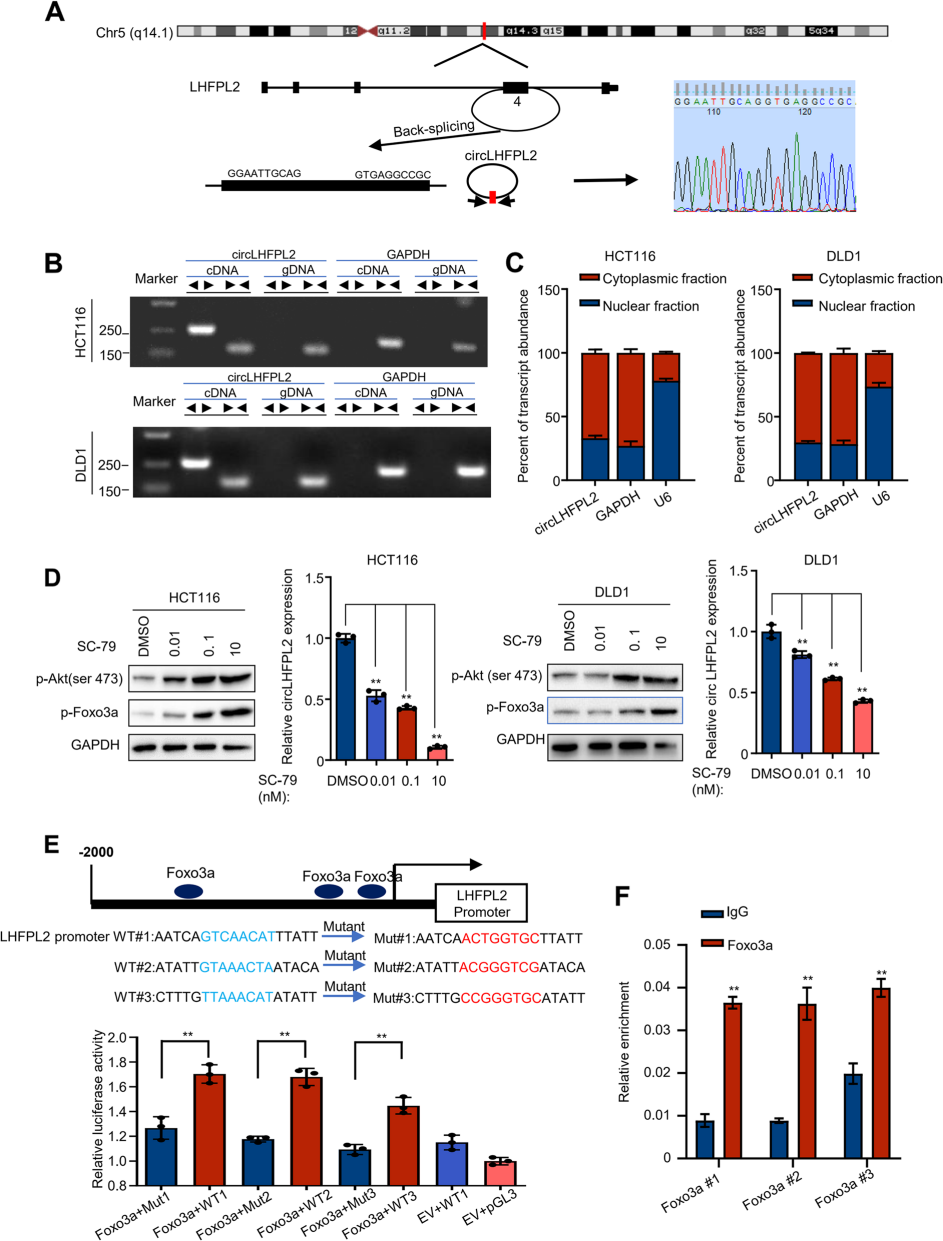
CircLHFPL2 was the target gene of the transcription factor Foxo3a. **A** Schematic illustration showing LHFPL2 exon 4 forming circLHFPL2. The existence of circLHFPL2 was proven by RT–PCR and its back-splicing junction was verified by Sanger sequencing. Red arrow indicates the special splicing junction of circLHFPL2. **B** The existence of circLHFPL2 determined in HCT116 and DLD1 cell lines by qRT-PCR. Divergent primers amplified circLHFPL2 in cDNA but not genomic DNA (gDNA). GAPDH was utilized as an internal control. **C** The subcellular fraction assay revealed subcellular location of circLHFPL2 in HCT116 and DLD1 cells. **D** Western blotting showed that SC-79 increased the protein expression level of p-AKT and p-Foxo3a in HCT116 (left) cells and DLD1 (right) cells. **E** Luciferase reporter assay confirmed the interaction between LHFPL2 promoter and Foxo3a. **F** Chip assay confirmed the interaction between LHFPL2 and Foxo3a. Data are presented as mean ± SEM; *n* ≥ 3. *p < 0.05; **p < 0.01

We next assessed the structure of circLHFPL2, which was derived from exon 4 of LHFPL2 and formed a 615 nt circular transcript. The back-spliced junction of circLHFPL2 was amplified and confirmed by Sanger sequencing, which was consistent with the CircBase database (http://www.circbase.org/) [23] (Fig. 2A). To confirm the head-to-tail splicing of circLHFPL2, convergent primers for LHFPL2 mRNA and special divergent primers for circLHFPL2 were designed. RNA and genomic DNA (gDNA) were isolated from HCT116 and DLD1 cells and PCR amplification was performed. The results showed that circLHFPL2 amplified by divergent primer was detected only in cDNA, but not detected in gDNA (Fig. 2B). Then, the cytoplasm and nuclei were fractioned. CircLHFPL2 was found to be enriched in the cytoplasmic fraction, but not in the nuclei (Fig. 2C).

As previously reported, PI3K/AKT signaling pathway was continuously activated in PIK3CA-mutant cells [7]. Therefore, we investigated whether circLHFPL2 downregulation in PIK3CA-mutant cells was regulated by the activation of PI3K/AKT signaling pathway. With the treatment of AKT agonist SC-79, the expression of p-AKT was significantly increased in HCT116 and DLD1 cells. Notably, circLHFPL2 and LHFPL2 expression in these two cell lines was statistically significantly decreased in a concentration-dependent manner (Fig. 2D, Supplementary Fig. 5B). We predicted the transcription factor of circLHFPL2 using [24] (http://jaspar.genereg.net/) and found that the promoter region of circLHFPL2 contained 11 binding sites with Foxo3a (Fig. 2E upper panel). Interestingly, SC-79 treatment remarkably elevated the level of p-Foxo3a, indicating that activated PI3K/AKT axis may suppress the expression of circLHFPL2 by phosphorylating the transcription factor, Foxo3a (Fig. 2D). Overexpression of Foxo3a could upregulate PTEN expression in HCT116 and DLD1 cells (Supplementary Fig. 8A and B). We investigated the interaction between circLHFPL2 and Foxo3a using luciferase and ChIP assays, and three predicted binding sites of circLHFPL2 promoter and Foxo3a were identified. Foxo3a significantly increased the luciferase activity of circLHFPL2-WT, while no significant change was detected in circLHFPL2-Mut (Fig. 2E). Consistent with this result, the ChIP assay showed that Foxo3a directly occupied these three regions on circLHFPL2 promoter (Fig. 2F). Collectively, these results suggest that circLHFPL2 is mainly expressed in the cytoplasm and is suppressed by activated PI3K/AKT via phosphorylation of Foxo3a.

### Overexpression of circLHFPL2 sabotages the viability of PIK3CA-mutant cells

**Fig. 3:**
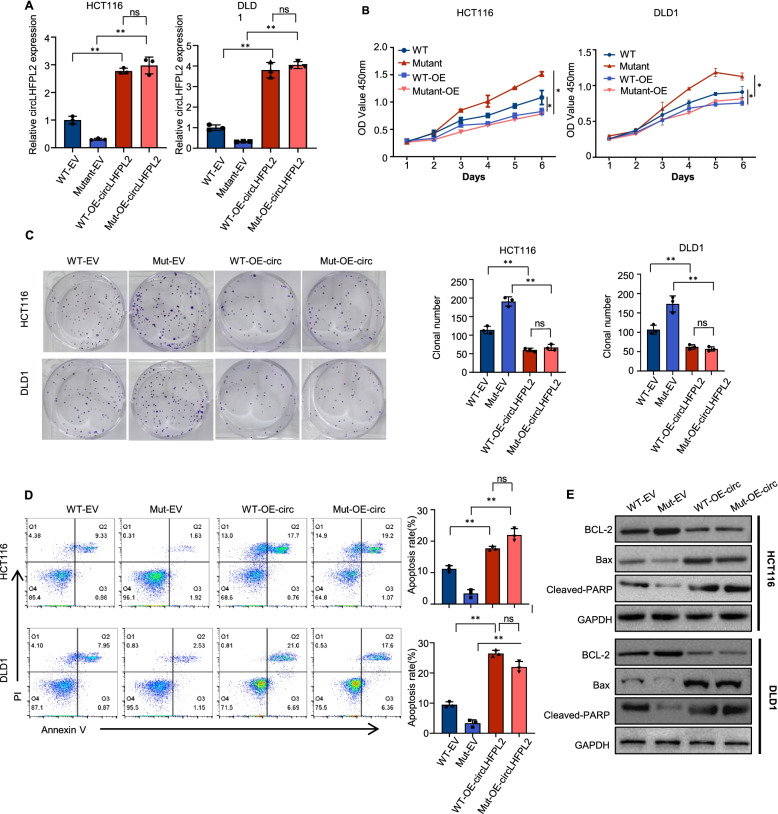
Overexpression of circLHFPL2 inhibited the growth of PIK3CA-mutant CRC cells. **A** Relative expression of circLHFPL2 determined via qRT-PCR in HCT116 PIK3CA^WT^ or PIK3CA^H1047R^ (left) and DLD1 PIK3CA^WT^ or PIK3CA^E545K^ cells (right) transfected with circLHFPL2 overexpression lentivirus. **B** CCK-8 assay revealed the viability of HCT116 PIK3CA^WT^ or PIK3CA^H1047R^ (left) and DLD1 PIK3CA^WT^ or PIK3CA^E545K^ cells (right) transfected with circLHFPL2 overexpression lentivirus. **C** Colony formation assay revealed the proliferation of HCT116 PIK3CA^WT^ or PIK3CA^H1047R^ (left) and DLD1 PIK3CA^WT^ or PIK3CA^E545K^ cells (right) transfected with circLHFPL2 overexpression lentivirus. **D** Apoptosis assay showed the apoptosis of HCT116 PIK3CA^WT^ or PIK3CA^H1047R^ (left) and DLD1 PIK3CA^WT^ or PIK3CA^E545K^ cells (right) transfected with circLHFPL2 overexpression lentivirus. **E** Western blotting showed the protein expression of BCL-2, Bax and cleaved PARP in HCT116 PIK3CA^WT^ or PIK3CA^H1047R^ (left) and DLD1 PIK3CA^WT^ or PIK3CA^E545K^ cells (right) transfected with circLHFPL2 overexpression lentivirus. Data are presented as mean ± SEM; *n* ≥ 3. *p < 0.05; **p < 0.01

**Fig. 4:**
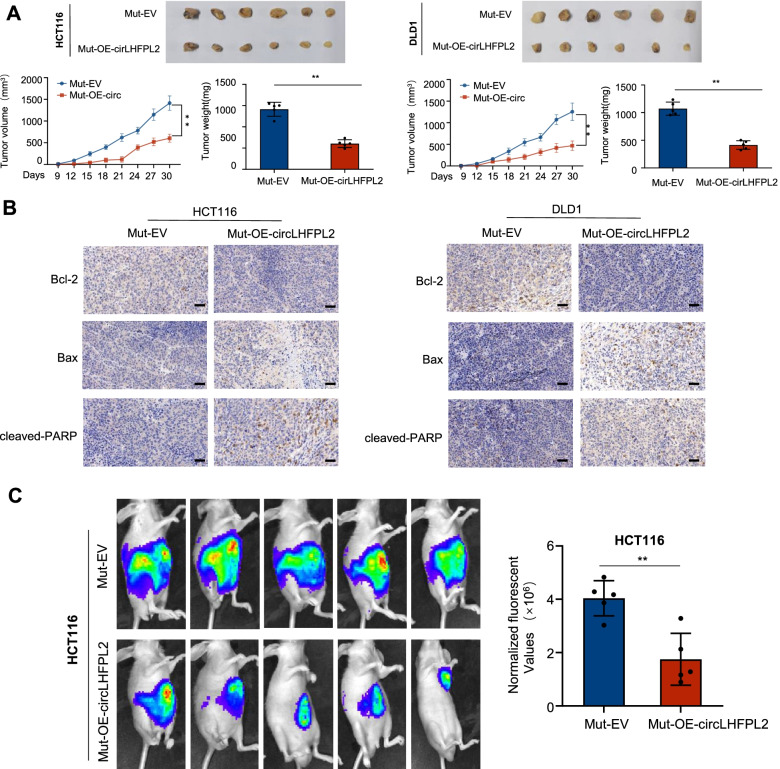
Overexpression of circLHFPL2 inhibited the growth of PIK3CA-mutant CRC xenograft tumor. **A** Treated cells (3 × 10^6^, 100 μL) were subcutaneously injected into the left flank of the mice. Tumor size was determined as previously reported (Length x Width^2^ × 0.5) every 3 days. After 30 days, the mice were sacrificed, and the tumor weight was measured. **B** IHC staining showed the protein expression of BCL-2, Bax, cleaved PARP in xenograft tumors. **C** A mouse model of lung metastases established by tail vein injection of the indicated HCT116 PIK3CA^H1047R^ and DLDL1 PIK3CA^E545K^ cells with circLHFPL2 overexpression. Representative bioluminescence images (**C**, left) and bioluminescence signals (**C**, right) acquired at 8 weeks after injection. Data are presented as mean ± SEM; *n* ≥ 3. *p < 0.05; **p < 0.01

Having demonstrated that the expression of circLHFPL2 was dramatically decreased in PIK3CA-mutant cells, we explored how the cells reacted to the overexpression of circLHFPL2. To this end, we overexpressed circLHFPL2 with lentivirus in HCT116 PIK3CA^WT^ or PIK3CA^H1047R^ and DLD1 PIK3CA^WT^ or PIK3CA^E545K^ cells (Fig. 3A). Then, we examined the effect of circLHFPL2 overexpression on cell proliferation using CCK-8 and colony formation assays. The results showed that overexpression of circLHFPL2 significantly suppressed cell viability, especially in PIK3CA^Mut^ cells (Fig. 3B and C). Moreover, apoptosis assays showed that circLHFPL2 overexpression promoted apoptosis in CRC cells, especially in PIK3CA Mut cells (Fig. 3D). Western blotting revealed that anti-apoptosis protein BCL-2 was downregulated, while apoptosis-associated proteins Bax and cleaved PARP were significantly upregulated (Fig. 3E). To further confirm the antitumor effect of circLHFPL2, we designed and synthesized two siRNAs (si-circLHFPL2–1 and si-circLHFPL2–2) targeting the back-splicing junction of circLHFPL2 to silence circLHFPL2 in HCT116 cells (Supplementary Fig. 3A). As shown in Supplementary Fig. 3B-C, silencing circLHFPL2 promotes HCT116 cell proliferation and inhibits cell apoptosis. Finally, we explored how circLHFPL2 affected the in vivo tumor growth using subcutaneous xenograft tumor model with PI3KCA Mut cells. The results showed that circLHFPL2 overexpression in HCT116 PIK3CA^H1047R^ and DLD1 PIK3CA^E545K^ cells significantly suppressed the growth of xenograft tumors (Fig. 4A). Moreover BCL-2 was downregulated while Bax and cleaved PARP were upregulated when circLHFPL2 was overexpressed (Fig. 4B). We detected the effect of linear LHFPL2 overexpression on HCT116 proliferation and found that overexpression of LHFPL2 have no effect on proliferation of HCT116 cells (Supplementary Fig. 5C-E). In the lung metastasis model, stable overexpression of circLHFPL2 in HCT116 PIK3CA^H1047R^ cells substantially inhibited tumor metastasis to the lung (Fig. 4C). Taken together, these results revealed that circLHFPL2 inhibits PI3KCA-mutant CRC progression both in vitro and in vivo.

### CircLHFPL2 acts as a ceRNA to sponge miR-556-5p and miR-1322

**Fig. 5:**
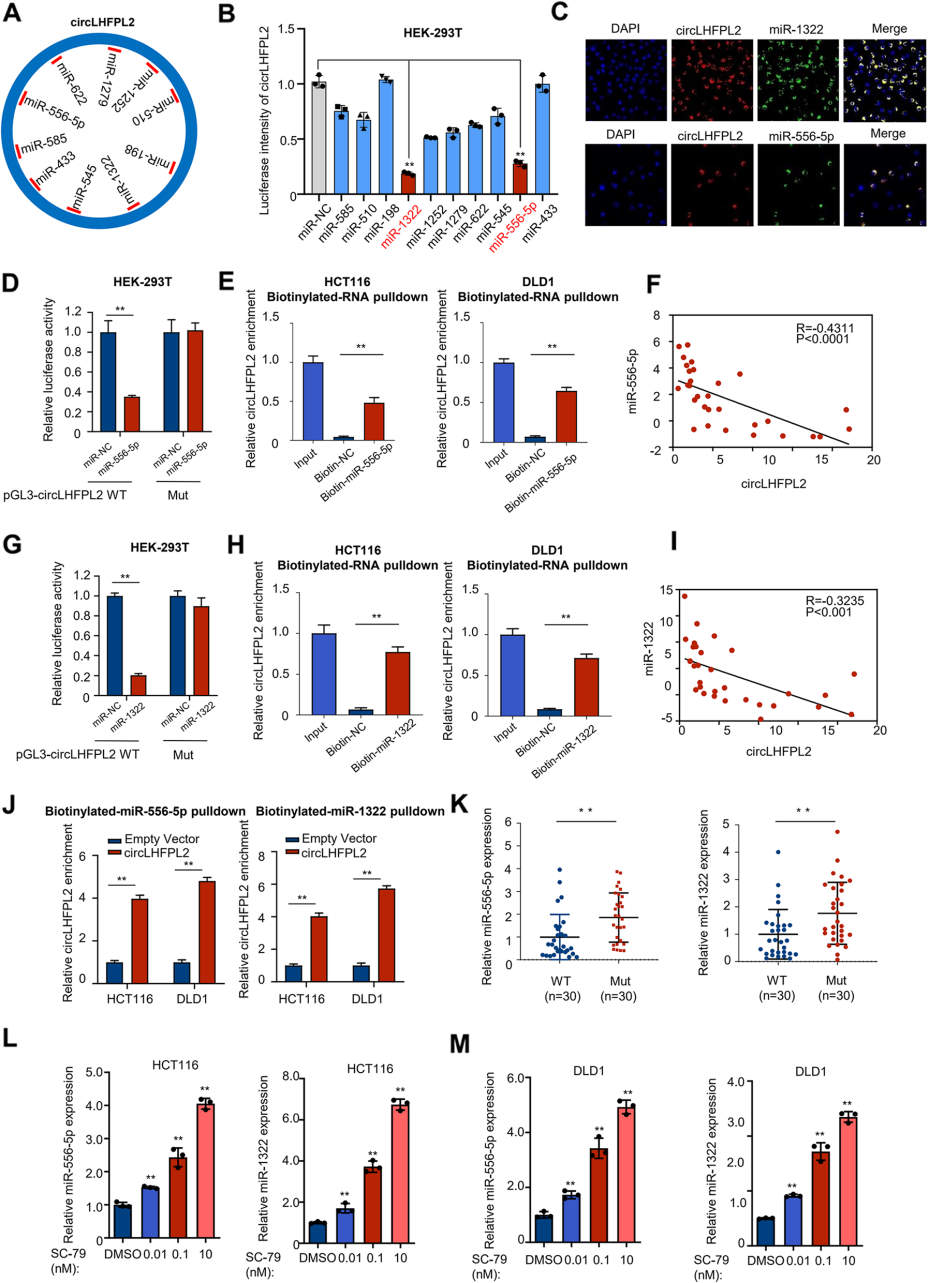
CircLHFPL2 acted as ceRNA to sponge miR-556-5p and miR-1322. **A** Schema of predicted miRNAs sponged by circLHFPL2 by using Circbase (https://starbase.sysu.edu.cn/) and Circinteractome (https://circinteractome.nia.nih.gov/). **B** Luciferase reporter assay confirmed the interaction between circLHFPL2 and predicted miRNAs. **C** Fluorescence in situ hybridization (FISH) examined the location of circLHFPL2 with miR-1322 and miR-556-5p. **D** and **G** Luciferase reporter assay confirmed the interaction between circLHFPL2 and miR-556-5p (**D**) or miR-1322 (**G**). **E** and **H** RNA pull down assay confirmed the interaction between circLHFPL2 and miR-556-5p (**E**) or miR-1322 (**H**). **F** and **I** Correlation between circLHFPL2 and miR-556-5p (**F**), circLHFPL2 and miR-1322 (**I**) analyzed by Pearson’s correlation analysis. **J** Binding of circLHFPL2 to 3′-end biotinylated miR-556-5p (left panel) and 3′-end biotinylated miR-1322 (right panel) in HCT116 cells with circTP63 overexpression. **K** Relative expression of miR-556-5p (left) and miR-1322 (right) was determined via qRT-PCR of tumor tissues from 30 CRC patients with PIK3CA Mut and 30 patients with PIK3CA WT. **L** and **M** qRT-PCR showed that SC-79 upregulated miR-556-5p and miR-1322 expression in HCT116 (**L**) and DLD1 (**M**) cells. Data are presented as mean ± SEM; *n* ≥ 3. *p < 0.05; **p < 0.01

Bioinformatics online analyses were utilized to predict the potential targets of circLHFPL2 by using Starbase and Circinteractome database. 10 predicted miRNAs related to cancer progression were selected for further analysis (Fig. 5A, Table S3). Dual-luciferase assays showed that miR-556-5p and miR-1322 were the potential downstream miRNAs binding to circLHFPL2 (Fig. 5B, supplementary Fig. 1A and B). Fluorescence in situ hybridization (FISH) showed that circLHFPL2 was localized in the cytoplasm, similar to mature miRNAs [22] (Fig. 5C). Dual-luciferase reporter and RNA pull-down assays were then implemented to confirm the interaction between circLHFPL2 and miR-556-5p and miR-1322. Cells co-transfected with pGL3-circLHFPL2 WT plasmid and miR-556-5p mimics exhibited reduced activity compared with that in the control groups (Fig. 5D). In line with this, biotinylated miR-556-5p probe effectively captured circLHFPL2 compared with control groups (Fig. 5E and J [left panel]). In addition, cells co-transfected with circLHFPL2 WT and miR-1322 mimics also showed decreased activity compared with that in the control groups (Fig. 5G). Similarly, biotinylated miR-1322 probe effectively captured circLHFPL2 relative to control groups (Fig. 5H and J [right panel]). We further evaluated the expression of miR-556-5p and miR-1322 in 30 CRC PIK3CA Mut tissues and 30 PIK3CA WT tissues. The results showed that miR-556-5p and miR-1322 were significantly upregulated in CRC PIK3CA-mutant tissues (Fig. 5K). qRT-PCR results showed that miR-556-5p and miR-1322 were upregulated in circLHFPL2-overexpressed transplant tumors (Supplementary Fig. 6B). We also detected miR-556-5p and miR-1322 expression after SC-79 treatment. As shown in Fig. 5L-M, miR-556-5p and miR-1322 expression in these two cell lines were statistically significantly increased in a concentration-dependent manner after SC-79 treatment. The correlation between circLHFPL2 and miR-556-5p/miR-1322 in patient tissues was analyzed. It was found that the expression of miR-556-5p or miR-1322 was negatively associated with circLHFPL2 abundance (Fig. 5F and I). Moreover, survival analysis revealed that patients with high expression of miR-1322 and miR-556-5p were significantly associated with poor overall survival (Supplementary Fig. 6C). We detected miR-1322 and miR-556-5p expression in HCT116 and DLD1 cells transfected with Foxo3a-overexpressing plasmid (Supplementary Fig. 8A), and found that overexpression of Foxo3a significantly inhibited miR-1322 and miR-556-5p expression (Supplementary Fig. 8C). These results revealed that circLHFPL2 acts as a ceRNA to sponge miR-556-5p and miR-1322 in CRC cells.

### PTEN is the downstream target gene of miR-556-5p and miR-1322

**Fig. 6:**
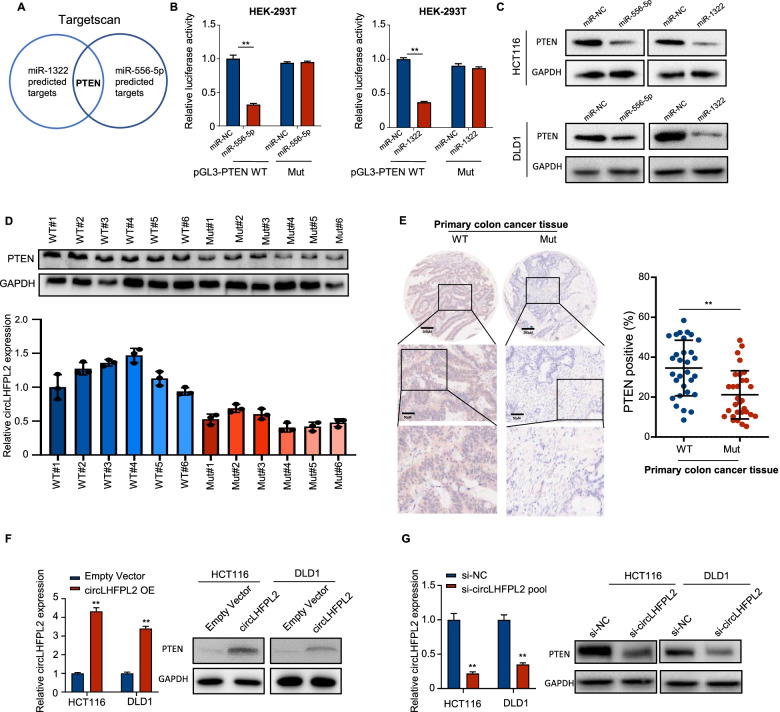
PTEN was the downstream target gene of miR-556-5p and miR-1322. **A** Veinn analysis of predicted targets of miR-556-5p and miR-1322 in Targetscan database. **B** Luciferase reporter confirmed the interaction between miR-556-5p and PTEN (left), miR-1322 and PTEN (right). **C** Protein level of PTEN reduced under overexpression of miR-556-5p and miR-1322 in HCT116 (Upper) and DLD1 (Lower) cells by Western blotting. **D **Relative expression of circLHFPL2 determined by qRT-PCR in tumor tissues of 5 CRC patients with PIK3CA Mut and 5 patients with PIK3CA WT. Relative expression of PTEN determined by Western blotting in tumor tissues of 5 CRC patients with PIK3CA Mut and 5 patients with PIK3CA WT. **E** Tissue microarray (TMA) analysis for PTEN expression in 30 PIK3CA-mutant CRC tissues and 30 PIK3CA non-mutant CRC tissues by IHC (Immunohistochemistry). **F** Relative expression of PTEN in HCT116 and DLD1 cells transfected with circLHFPL2 overexpression plasmid. **G** Relative expression of PTEN in HCT116 and DLD1 cells transfected with circLHFPL2 siRNAs. Data are presented as mean ± SEM; n ≥ 3. *p < 0.05; **p < 0.01

MiR-556-5p and miR-1322 play a promoting role in tumorigenesis of several types of cancers [25–27]. In line with this, our results showed the upregulation of miR-556-5p and miR-1322 in PI3KCA Mut CRC. We further explored the downstream targets of miR-556-5p and miR-1322 using Targetscan databases. PTEN was predicted as a possible downstream target of miR-556-5p and miR-1322 (Fig. 6A and Supplementary Fig. 1B and C). Dual-luciferase reporter assay was carried out to validate the association between miR-556-5p or miR-1322 and PTEN. The results showed that cells co-transfected with pGL3-PTEN WT plasmid and miR-556-5p mimics exhibited reduced activity compared with that in the control groups in 293 T cells (Fig. 6B left panel). Similarly, cells co-transfected with PTEN WT and miR-1322 mimics also exhibited lower activity relative to that in the control groups in 293 T cells (Fig. 6B right panel). Notably, protein levels of PTEN were significantly reduced after overexpression of miR-556-5p or miR-1322 in HCT116 and DLD1 cells (Fig. 6C, Supplementary Fig. 7A). Six CRC PIK3CA-mutant tissues and six PIK3CA WT tissues were selected to evaluate the expression of PTEN and circLHFPL2. The results showed a positive correlation between circLHFPL2 and PTEN expression, and both were significantly downregulated in CRC PIK3CA Mut tissues (Fig. 6D). Then, IHC staining was used to confirm the expression of PTEN in CRC PIK3CA WT and Mut tissues via TMA. The results showed that PTEN is downregulated in PIK3CA-mutant tissues (Fig. 6E). To further explore whether PTEN expression could be regulated by circLHFPL2, we measured the expression of PTEN in circLHFPL2-overexpressed cells. The results showed that PTEN protein levels were significantly upregulated in HCT116 and DLD1 cells with upregulation of circLHFPL2 (Fig. 6F), but when circLHFPL2 was silenced, its levels were downregulated (Fig. 6G). Overexpression of Foxo3a inhibited PTEN expression in HCT116 and DLD1 cells (Supplementary Fig. 8D). These results showed that circLHFPL2 upregulates PTEN expression by sponging miR-556-5p and miR-1322.

### Downregulation of circLHFPL2 sustains the activation of PI3K/AKT signaling pathway by forming a regulatory loop with miR-556-5p/miR-1322/PTEN axis

**Fig. 7:**
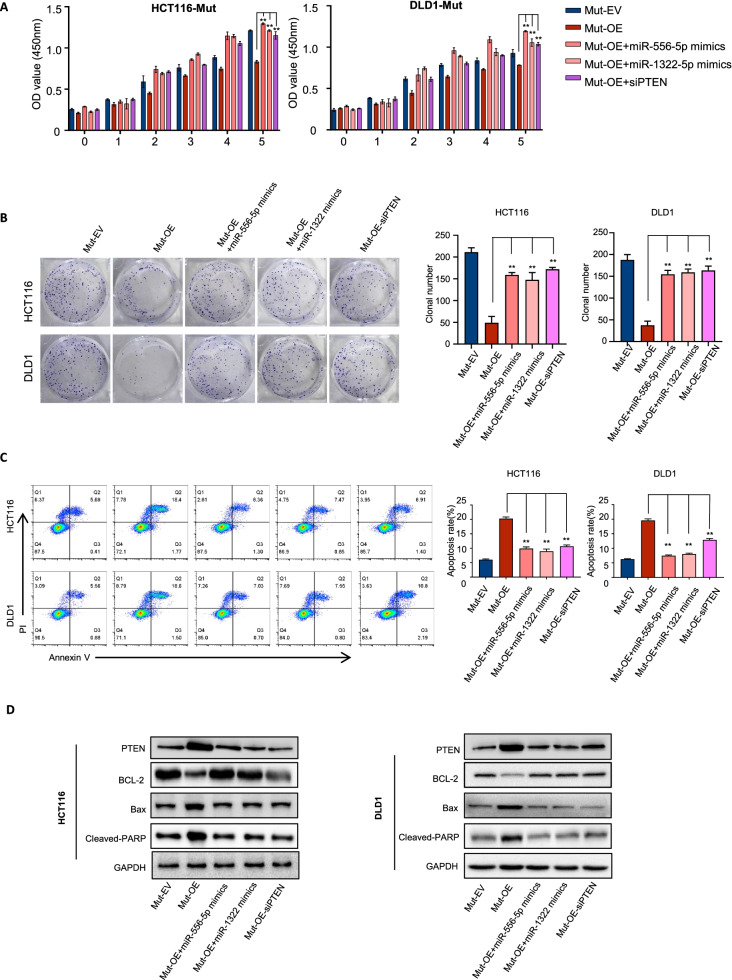
Downregulation of circLHFPL2 sustained activation of PI3K/AKT pathway via a positive feedback loop of miR-556-5p and miR-1322/PTEN axis. **A** CCK-8 assay on the viability of PIK3CA^H1047R^ and PIK3CA^E545K^ cells transfected with circLHFPL2 overexpression plasmid or with the combination of miR-556-5p mimics, miR-1322mimics and siPTEN. **B** Colony formation assay on the proliferation of PIK3CA^H1047R^ and PIK3CA^E545K^ cells transfected with circLHFPL2 overexpression plasmid or with the combination of miR-556-5p mimics, miR-1322mimics and siPTEN. **C** Apoptosis assay showed the apoptosis of PIK3CA^H1047R^ and PIK3CA^E545K^ cells transfected with circLHFPL2 overexpression plasmid or with the combination of miR-556-5p mimics, miR-1322mimics and siPTEN. **D** Western blotting showed the protein expression level of PTEN, BCL-2, Bax and cleaved PARP in PIK3CA^H1047R^ and PIK3CA^E545K^ cells transfected with circLHFPL2 overexpression plasmid or with the combination of miR-556-5p mimics, miR-1322mimics and siPTEN. Data are presented as mean ± SEM; *n* ≥ 3. *p < 0.05; **p < 0.01

To further verify whether circLHFPL2 downregulation could activate the PI3K/AKT pathway via a regulatory loop with the miR-556-5p/miR-1322/PTEN axis, we performed a series of rescue experiments. CCK-8 and colony formation assays revealed that the cell viability of HCT116 PIK3CA^H1047R^ and DLD1 PIK3CA^E545K^ cells was significantly inhibited after circLHFPL2 overexpression, while the cell viability was partially recovered in the presence of a combination of miR-556-5p or miR-1322 mimics or siPTEN (Fig. 7A and B, Supplementary Fig. 7B). Moreover, circLHFPL2 overexpression accelerated cell apoptosis, while the combination of miR-556-5p or miR-1322 mimics or siPTEN partially inhibited apoptosis in the HCT116 PIK3CA^H1047R^ and DLD1 PIK3CA^E545K^ cells (Fig. 7C). We further investigated the apoptosis-associated proteins involved in this biological process. The results showed that circLHFPL2 overexpression significantly decreased anti-apoptosis protein BCL-2 and increased PTEN, as well as apoptosis-associated proteins Bax and cleaved PARP, while these effects were partially recovered with the combination of miR-556-5p/miR-1322mimics or siPTEN (Fig. 7D). We isolated primary cells from a colon cancer tissue harboring PIK3CA E545K mutation and further confirmed that circLHFPL2 inhibits CRC progression by downregulating PTEN (Supplementary Fig. 2). Collectively, these results indicated that downregulation of circLHFPL2 sustains activation of PI3K/AKT pathway by regulating miR-556-5p/miR-1322/PTEN axis.

### Overexpression of CircLHFPL2 overcomes MEK inhibition resistance in CRC with PIK3CA mutation

**Fig. 8:**
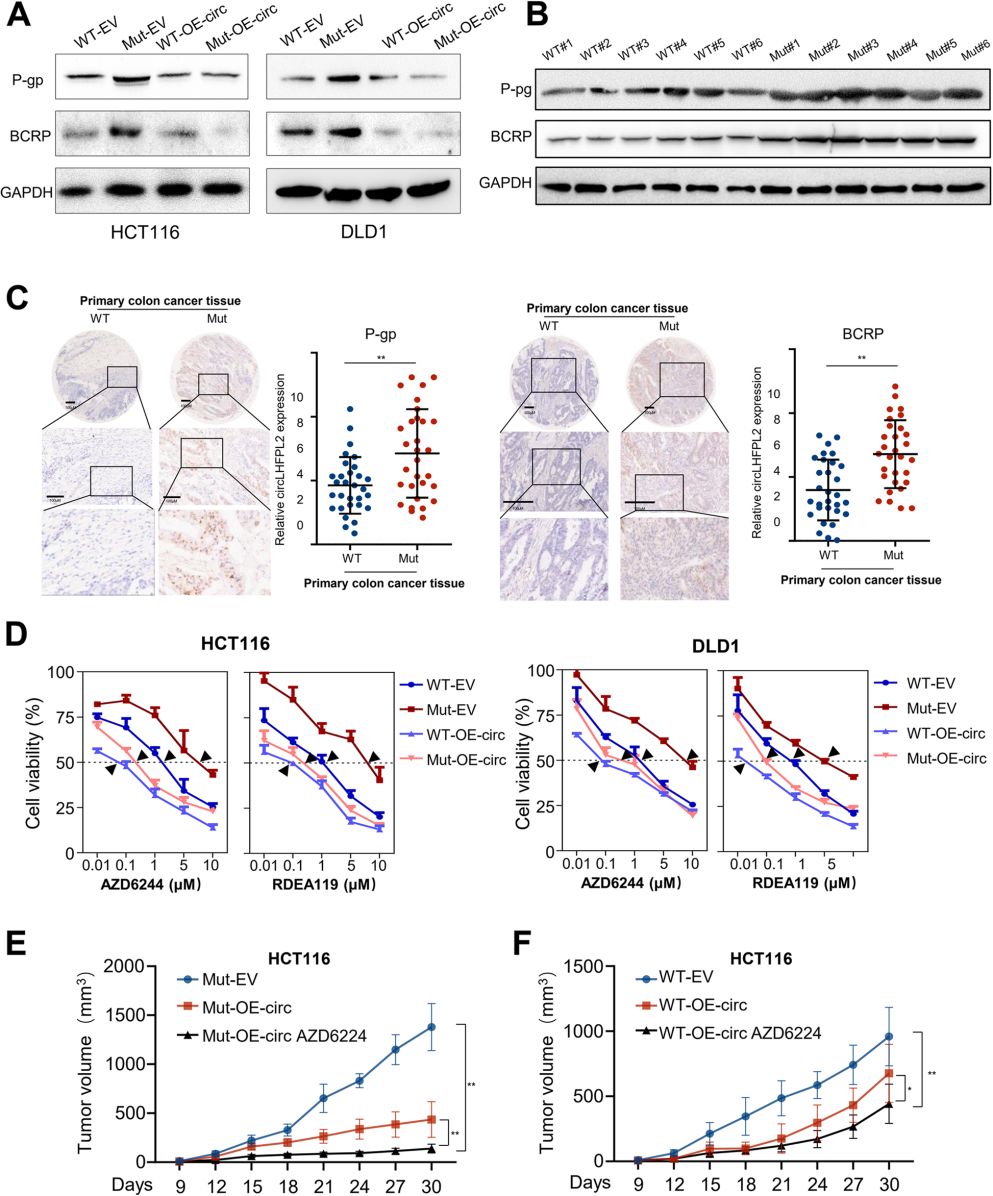
Overexpression of circLHFPL2 improved sensitivity of MEK inhibitors in CRC cells with PIK3CA mutations. **A** Western blotting showed the protein expression level of P-gp and BCRP in HCT116 and DLD1 cells with or without PIK3CA mutation, transfected with circLHFPL2 overexpression plasmid or empty vector. **B** Western blotting showed the protein expression of P-gp and BCRP in CRC tissues with or without PIK3CA mutations. **C** Tissue microarray (TMA) analysis for P-gp and BCRP expression in 30 PIK3CA-mutant and 30 PIK3CA non-mutant CRC tissues by immunohistochemistry. **D** CCK-8 assay revealed the viability of PIK3CA^H1047R^ and PIK3CA^E545K^ cells transfected with circLHFPL2 overexpression plasmid or with empty vector treatment with AZD6244 or RDEA119 for 24 h (inhibitor concentration: 0.01, 0.1, 1, 5, 10 μM). Black arrow indicates IC50 value. **E** and **F** AZD6244 inhibited the growth of xenografted tumors of HCT116 PIK3CA^H1047R^ (**E**) and WT (**F**) cells. Overexpression of circLHFPL2 enhanced the tumor sensitivity to AZD6244 especially in PIK3CA-mutant tumors. 10 mg/kg of AZD6244 was injected intraperitoneally every 3 days. Data are presented as mean ± SEM; *n* ≥ 3

In our previous study, we found that targeting PI3K overcomes P-gp- and BCRP-mediated cancer multidrug resistance (MDR) in cancers [11]. PI3K activation leads to P-gp and BCRP overexpression and decreases the sensitivity of colon cancer cells to a MEK inhibitor [12]. As shown in the above data, overexpression of circLHFPL2 decreased activation of PI3K by upregulating PTEN, suggesting that circLHFPL2 may be a potential target that could overcome P-gp- and BCRP-mediated MDR of colon cancer with PIK3CA mutation. Thus, P-gp and BCRP expression in colon cancer cells with PIK3CA mutation was detected by Western blotting analysis. As shown in Fig. 8A, P-gp and BCRP were upregulated in HCT116 PIK3CA^H1047R^ and DLD1 PIK3CA^E545K^ cells compared with PIK3CAWT cells, whereas P-gp and BCRP were decreased in circLHFPL2 overexpression HCT116 and DLD1, especially in PIK3CA-mutant cells. Moreover, the protein levels of P-gp and BCRP were found to be significantly higher in colon cancer tissues with PIK3CA mutations than in PIK3CA WT tissues (Fig. 8B-C). Colon cancer cells with or without PIK3CA mutation and with or without circLHFPL2 overexpression were treated with MEK inhibitors AZD6244 or RDEA119 for 24 h. Compared to the HCT116 and DLD1 PIK3CA-mutant cells, the cell viability of the WT cells was greatly reduced upon treatment with AZD6244 or RDEA119, indicating that PIK3CA mutation enhanced drug resistance of colon cancer cells (Fig. 8D). Interestingly, HCT116 and DLD1 cell lines with circLHFPL2 overexpression were more sensitive to AZD6244 and RDEA119, especially in PIK3CA-mutant cells (Fig. 8D). Moreover, overexpression of circLHFPL2 enhanced the antitumor effect of AZD6244 in PIK3CA-mutant tumors (Fig. 8E-F). These results suggest that PIK3CA mutation enhanced MEK inhibitor resistance, which can be reversed by circLHFPL2.

## Discussion

PIK3CA is one of the most frequently mutated oncogenic genes and plays an important role in tumorigenesis and drug resistance [5]. Recent studies have demonstrated that mutant PIK3CA can activate the PI3K/AKT signaling pathway without activating growth factors [7]. Samuels et al. demonstrated that PI3K/AKT signaling pathway was activated in cells with mutant PIK3CA at 1% FBS concentration, but was not activated in cells with WT PIK3CA [6]. Furthermore, mutant PIK3CA could gain functions independent of growth factor stimulation, by directly binding to IRS1 [7]. In this study, we revealed a novel regulatory network involving the activation of AKT and its downstream targets in a growth factor-independent manner in mutant PIK3CA CRC. Through circRNA sequencing, we identified that circLHFPL2 was downregulated in both HCT116 PIK3CA^H1047R^ and DLD1 PIK3CA^E545K^ cell lines, which was also observed in PIK3CA-mutant CRC primary cells and tissues. The downregulation of circLHFPL2 was correlated with poor prognosis. Furthermore, we found that circLHFPL2 downregulation was induced by activation of PI3K/AKT signaling pathway caused by PIK3CA mutation mediated by FOXO3a phosphorylation, which in turn sustained the activation of PI3K/AKT signaling pathway via a regulatory loop with miR-556-5p/miR-1322/PTEN axis. In addition, our data demonstrate that circLHFPL2 promotes the sensitivity of CRC cells to MEK inhibitors by downregulating the expression of P-gp and BCRP, particularly in PIK3CA-mutant cells. To the best of our knowledge, this study for the first time revealed a novel mechanism linking circRNA and mutant PI3KCA in continuously activating AKT signaling in a growth factor-independent manner. More importantly, we identified circLHFPL2 as a new biomarker for MEK inhibitor therapy, which has important clinical implications for the treatment of CRC with PIK3CA mutation [28]. Low circHPFPL2 expression is related to poor prognosis of CRC patients, and therefore an independent external cohort needs to further validate that circLHFPL2 could serve as a prognostic biomarker for CRC diagnosis.

CircLHFPL2 is a 615 nt circular transcript originated from exon 4 of LHFPL2. LHFPL2 is a member of the lipoma high mobility group protein isoform I-C (HMGIC) fusion partner (LHFP) gene family, which encodes a tetra-transmembrane protein [29]. LHFPL2 has been implicated in diverse physiological functions, including long-term proliferation of leukemic cells, coronary heart disease and infertility [29–31]. The expression profiles and functional roles of LHFPL2-formed circRNA on CRC progression have not yet been reported. CircLHFPL2 overexpression suppressed CRC cell proliferation and weakened the viability of PIK3CA-mutant cells, indicating that circLHFPL2 plays a crucial role in the development of PIK3CA-mutant tumors.

We also revealed the mechanism of circLHFPL2 downregulation in CRC with PIK3CA mutations. FOXO3a belongs to the FOXO subfamily that acts as a forkhead transcription factor regulating various biological processes, such as proliferation, apoptosis, cell cycle and tumorigenesis [32]. Growing evidence supports that FOXO3a acts as a tumor suppressor in cancers by regulating downstream signaling targets through transcriptional modification [33, 34]. FOXO3a inactivation is observed in various cancers and is mainly caused by mutation of FOXO3a gene or cytoplasmic sequestration of FOXO3a protein induced by post-translational modification [32]. Foxo3a could be phosphorylated by activated PI3K/AKT signaling pathway, and the phosphorylated Foxo3a can bind to 14–3-3 protein, which leads to nuclear export and ubiquitination of Foxo3a, and thus inhibiting the transcription of target genes [35]. Here, we identified Foxo3a as the transcription factor of circLHFPL2. Consistent with the previous study, we found that Foxo3a maintains a high phosphorylation state in cells with PIK3CA mutations, which is caused by persistently activated Akt, and explains how PIK3CA mutation downregulates circLHFPL 2 [36].

PTEN, namely phosphatase and tensin homologue, is an important tumor suppressor in various cancers including ovarian cancer, melanoma, gastric cancer, breast cancer, prostate cancer and colorectal cancer. PTEN inhibits proliferation and promotes apoptosis of cancer cells [37]. PTEN dephosphorylates PIP3 to PIP2, thus abrogating PI3K/AKT activity and inhibiting the progression of cancers [38, 39]. In cancer cells, PTEN is inactivated by multiple mechanisms, including mutations, promoter hypermethylation, protein degradation and subcellular mislocalization [39]. Although PTEN is demonstrated to modulate PI3K/AKT activity, the regulatory role of mutant PIK3CA on PTEN remains an enigma. Here, we revealed a novel regulatory mechanism of mutant PIK3CA on PTEN. We found that circLHFPL2 could sponge miR-556-5p and miR-1322 in PIK3CA-mutant cells, thus regulating the expression of PTEN. Collectively, circLHFPL2 downregulation sustained the activation of PI3K/Akt signaling pathway via a regulatory loop with miR-556-5p/miR-1322/PTEN axis in PI3KCA mutant CRC.

Despite considerable efforts, the clinical outcome of treatments for solid tumors has been challenging [40–42]. Reasons include drug resistance such as that resulting from the dysregulation PI3K/Akt signaling pathway and PTEN suppression, and more. As reported, activated PI3K leads to P-gp and BCRP expression and increases the sensitivity of CRC cells to MEK inhibitor [11, 12]. Interestingly, in our study, overexpression of circLHFPL2 could inhibit the expression of P-gp and BCRP and overcome PI3KCA mutation-mediated MEK inhibitor resistance in CRC cells. In our previous study, we found that PI3K promoted drug resistance by upregulating P-gp and BCRP in human breast cancer [11]. Thus, we assumed that circLHFPL2 may inhibit P-gp and BCRP expression by regulating PI3K. Our study indicated that circLHFPL2 may serve as a potential therapy target for regulating PIK3CA mutation-mediated drug resistance. However, limited by the in vivo and in vitro models we used, the function of circLHFPL2 on regulating MEK inhibitor in the treatment of CRC patients needs to be further investigated from the perspective of clinical treatment in the future.

## Conclusion

**Fig. 9:**
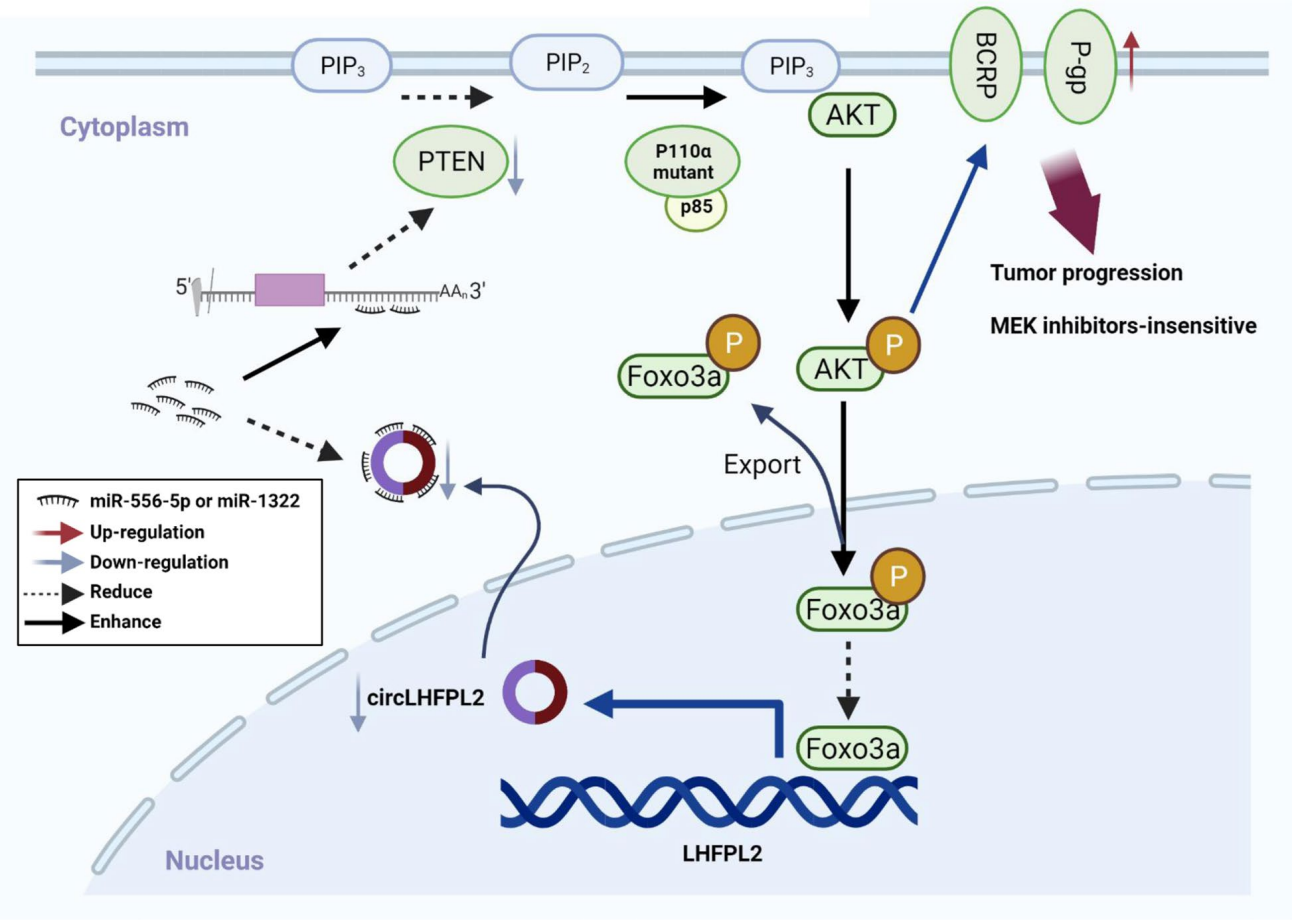
Schema of the positive feedback loop of sustained activation of PI3K/AKT via circLHFPL2/miR-556-5p and miR-1322/PTEN axis and downregulation of circLHFPL2 enhanced MEK inhibitor resistance by upregulating P-gp and BCRP in PIK3CA-mutant CRC cells

In summary, our study revealed a new mechanism underlying PIK3CA mutation in promoting cancer progression, as well as the function of circLHFPL2 regulated by PI3K/Akt signaling pathway in CRC. In addition, we demonstrated that circLHFPL2 regulates the sensitivity of CRC cells to MEK inhibitor and could be a biomarker for MEK inhibitor treatment in patients whose tumors harbor PIK3CA mutations (Fig. 9). Future experiments may extend our study to a large panel of CRC cell lines, patient-derived xenografts and transgenic PIK3CA-mutant mouse models.

## Supplementary Information

**Additional file 1: Table S1.** Primers and DNA/RNA srquence used in this study.

**Additional file 2: Table S2.** Antibody information.

**Additional file 3: Table S3.** The list of predicted miRNAs.

**Additional file 4: Supplementary Fig. 1.** Predicted binding sites. (A) Predicted binding site between circLHFPL2 and miR-556-5p. (B) Predicted binding site between circLHFPL2 and miR-1322. (C) Predicted binding site between PTEN and miR-556-5p. (D) Predicted binding site between PTEN and miR-1322.

**Additional file 5: Supplementary Fig. 2.** Overexpression of circLHFPL2 inhibits primary colon cancer cell proliferation and promotes apoptosis by regulating PTEN. (A) circLHFPL2 expression detected by qRT-PCR in cells transfected with circLHFPL2 overexpression plasmid or co-transfected with si-PTEN (left panel). PTEN expression was detected by Western blot in cells transfected with circLHFPL2 overexpression plasmid or co-transfected with si-PTEN (right panel). (B) CCK8 assays examined the proliferation of cells transfected with circLHFPL2 overexpression plasmid or co-transfected with si-PTEN. (C) Apoptosis assays examined the apoptosis rate of cells transfected with circLHFPL2 overexpression plasmid or co-transfected with si-PTEN. Data are presented as mean ± SEM; *n* ≥ 3. *p < 0.05; **p < 0.01.

**Additional file 6: Supplementary Fig. 3.** Silencing circLHDPL2 promotes the viability of HCT116 cells. (A) circLHFPL2 expression detected by qRT-PCR after siRNA which targets circLHFPL2 in HCT116 cells. (B) CCK8 assay was used to examine the proliferation of HCT116 cells transfected with siRNA which targets circLHFP2L. (C) Apoptosis assay was used to examine the apoptosis of HCT116 cells transfected with siRNA which targets circLHFP2L. Data are presented as mean ± SEM; *n* ≥ 3. *p < 0.05; **p < 0.01.

**Additional file 7: Supplementary Fig. 4.** The expression of circLHFPL2 in HCT116 mutant and WT clones normalized to GAPDH and β-actin.

**Additional file 8: Supplementary Fig. 5.** Overexpression of linear LHFPL2 has no effect on HCT116 cell viability. (A) Linear LHFPL2 expression in 30 PIK3CA-mutant and 30 WT CRC tissues. (B) Linear LHFP2 was downregulated upon SC-79 treatment. (C) qRT-PCR detected the expression of linear LHFPL2 expression in HCT116 cells transfected with LHFPL2 overexpression plasmids. (D) CCK8 assay examined the proliferation of HCT116 cells transfected with LHFPL2 overexpression plasmids. (E) Apoptosis assay examined the apoptotic rate of HCT116 cells transfected with LHFPL2 overexpression plasmids. Data are presented as mean ± SEM; *n* ≥ 3. *p < 0.05; **p < 0.01.

**Additional file 9: Supplementary Fig. 6.** (A) Relative quantification of Bcl-2, Bax, cleaved PARP for Fig. 2B. (B) miR-556-5p and miR-1322 expression in xenografted tumors. (C) Survival analysis in patients with different expression pattern of miR-556-5p (left) and miR-1322 (right) with Kaplan-Meier method. Data are presented as mean ± SEM; *n* ≥ 3. *p < 0.05; **p < 0.01.

**Additional file 10: Supplementary Fig. 7.** The transfection efficiency of miR-556-5p mimics (A, left), miR-1322 mimics (A, right) and siRNA which targets PTEN (B). Data are presented as mean ± SEM; *n* ≥ 3. *p < 0.05; **p < 0.01.

**Additional file 11: Supplementary Fig. 8.** The effect of overexpression of FOXO3a on circLHFPL2, miR-556-5p, miR-1322 and PTEN expression. (A) Western blot detected the FOXO3a expression in HCT116 and DLD1 cells transfected with FOXO3a overexpression plasmids. (B) qRT-PCR detected the circLHFPL2 expression in HCT116 and DLD1 cells transfected with FOXO3a overexpression plasmids. (C) qRT-PCR detected the miR-556-5p and miR-1322 expression in HCT116 and DLD1 cells transfected with FOXO3a overexpression plasmids. (D) Western blot detected the PTEN expression in HCT116 and DLD1 cells transfected with FOXO3a overexpression plasmids.

## Data Availability

For all data requests, please contact the corresponding author.

## Abbreviations

CRC: Colorectal cancer
PIK3CA: Phosphatidylinositol-4,5-bisphosphate 3-kinase catalytic subunit alpha
PI3K: Class I phosphoinositide 3-kinase
PTEN: Phosphatase and tensin homolog
LHFPL2: LHFPL tetraspan subfamily member 2
circRNAs: Circular RNAs; miRNA: microRNA
qRT-PCR: Quantitative RT-PCR
FISH: Fluorescence in situ hybridization
MEK: Mitogen-activated protein kinase
ceRNA: Competing endogenous RNAs
BCRP/ABCG2: Breast cancer resistance protein
P-gp /ABCB1: P-glycoprotein
TMA: Tissue microarray

## References

[1] Czech MP (2000). PIP2 and PIP3: complex roles at the cell surface. Cell.

[2] Arendt KL, Royo M, Fernandez-Monreal M, Knafo S, Petrok CN, Martens JR, Esteban JA (2010). PIP3 controls synaptic function by maintaining AMPA receptor clustering at the postsynaptic membrane. Nat Neurosci.

[3] Cantley LC (2002). The phosphoinositide 3-kinase pathway. Science.

[4] Ashworth A, Hudson TJ (2013). Genomics: comparisons across cancers. Nature.

[5] Burke JE, Perisic O, Masson GR, Vadas O, Williams RL (2012). Oncogenic mutations mimic and enhance dynamic events in the natural activation of phosphoinositide 3-kinase p110alpha (PIK3CA). Proc Natl Acad Sci U S A.

[6] Samuels Y, Diaz LA, Schmidt-Kittler O, Cummins JM, Delong L, Cheong I, Rago C, Huso DL, Lengauer C, Kinzler KW (2005). Mutant PIK3CA promotes cell growth and invasion of human cancer cells. Cancer Cell.

[7] Hao Y, Wang C, Cao B, Hirsch BM, Song J, Markowitz SD, Ewing RM, Sedwick D, Liu L, Zheng W, Wang Z (2013). Gain of interaction with IRS1 by p110alpha-helical domain mutants is crucial for their oncogenic functions. Cancer Cell.

[8] Pearson HB, Li J, Meniel VS, Fennell CM, Waring P, Montgomery KG, Rebello RJ, Macpherson AA, Koushyar S, Furic L (2018). Identification of Pik3ca mutation as a genetic driver of prostate Cancer that cooperates with Pten loss to accelerate progression and castration-resistant growth. Cancer Discov.

[9] Saito Y, Koya J, Araki M, Kogure Y, Shingaki S, Tabata M, McClure MB, Yoshifuji K, Matsumoto S, Isaka Y (2020). Landscape and function of multiple mutations within individual oncogenes. Nature.

[10] Zhang M, Jang H, Nussinov R (2020). PI3K inhibitors: review and new strategies. Chem Sci.

[11] Zhang L, Li Y, Wang Q, Chen Z, Li X, Wu Z, Hu C, Liao D, Zhang W, Chen ZS (2020). The PI3K subunits, P110alpha and P110beta are potential targets for overcoming P-gp and BCRP-mediated MDR in cancer. Mol Cancer.

[12] Moon JH, Hong SW, Kim JE, Shin JS, Kim JS, Jung SA, Ha SH, Lee S, Kim J, Lee DH (2019). Targeting beta-catenin overcomes MEK inhibition resistance in colon cancer with KRAS and PIK3CA mutations. Br J Cancer.

[13] Kristensen LS, Andersen MS, Stagsted LVW, Ebbesen KK, Hansen TB, Kjems J (2019). The biogenesis, biology and characterization of circular RNAs. Nat Rev Genet.

[14] Cheng Z, Yu C, Cui S, Wang H, Jin H, Wang C, Li B, Qin M, Yang C, He J (2019). circTP63 functions as a ceRNA to promote lung squamous cell carcinoma progression by upregulating FOXM1. Nat Commun.

[15] Yu J, Xu QG, Wang ZG, Yang Y, Zhang L, Ma JZ, Sun SH, Yang F, Zhou WP (2018). Circular RNA cSMARCA5 inhibits growth and metastasis in hepatocellular carcinoma. J Hepatol.

[16] Zhang S, Chen Z, Sun J, An N, Xi Q (2020). CircRNA hsa_circRNA_0000069 promotes the proliferation, migration and invasion of cervical cancer through miR-873-5p/TUSC3 axis. Cancer Cell Int.

[17] Bian L, Zhi X, Ma L, Zhang J, Chen P, Sun S, Li J, Sun Y, Qin J (2018). Hsa_circRNA_103809 regulated the cell proliferation and migration in colorectal cancer via miR-532-3p / FOXO4 axis. Biochem Biophys Res Commun.

[18] Zeng K, Chen X, Xu M, Liu X, Hu X, Xu T, Sun H, Pan Y, He B, Wang S (2018). CircHIPK3 promotes colorectal cancer growth and metastasis by sponging miR-7. Cell Death Dis.

[19] Zhou C, Liu HS, Wang FW, Hu T, Liang ZX, Lan N, He XW, Zheng XB, Wu XJ, Xie D (2020). circCAMSAP1 promotes tumor growth in colorectal Cancer via the miR-328-5p/E2F1 Axis. Mol Ther.

[20] Li S, Zhang K, Khurshid M, Fan Y, Xu B, Zhou M. First report of Rhizoctonia solani AG-4 HGI causing stem canker on Fagopyrum tataricum (Tartary buckwheat) in China. Plant Dis. 2020.

[21] Rio DC, Ares M, Hannon GJ, Nilsen TW (2010). Purification of RNA using TRIzol (TRI reagent). Cold Spring Harb Protoc.

[22] Zhan Y, Zheng N, Teng F, Bao L, Liu F, Zhang M, Guo M, Guo W, Ding G, Wang Q (2017). MiR-199a/b-5p inhibits hepatocellular carcinoma progression by post-transcriptionally suppressing ROCK1. Oncotarget.

[23] Glazar P, Papavasileiou P, Rajewsky N (2014). circBase: a database for circular RNAs. RNA.

[24] Fornes O, Castro-Mondragon JA, Khan A, van der Lee R, Zhang X, Richmond PA, Modi BP, Correard S, Gheorghe M, Baranasic D (2020). JASPAR 2020: update of the open-access database of transcription factor binding profiles. Nucleic Acids Res.

[25] Zhang T, Zhao D, Wang Q, Yu X, Cui Y, Guo L, Lu SH (2013). MicroRNA-1322 regulates ECRG2 allele specifically and acts as a potential biomarker in patients with esophageal squamous cell carcinoma. Mol Carcinog.

[26] Zhang Y, Yu R, Li Q, Li Y, Xuan T, Cao S, Zheng J (2020). SNHG1/miR-556-5p/TCF12 feedback loop enhances the tumorigenesis of meningioma through Wnt signaling pathway. J Cell Biochem.

[27] Zhao W, Cao L, Zeng S, Qin H, Men T (2015). Upregulation of miR-556-5p promoted prostate cancer cell proliferation by suppressing PPP2R2A expression. Biomed Pharmacother.

[28] Liu P, Cheng H, Roberts TM, Zhao JJ (2009). Targeting the phosphoinositide 3-kinase pathway in cancer. Nat Rev Drug Discov.

[29] Zhao F, Zhou J, Li R, Dudley EA, Ye X (2016). Novel function of LHFPL2 in female and male distal reproductive tract development. Sci Rep.

[30] Hatfield KJ, Reikvam H, Bruserud O (2014). Identification of a subset of patients with acute myeloid leukemia characterized by long-term in vitro proliferation and altered cell cycle regulation of the leukemic cells. Expert Opin Ther Targets.

[31] Shendre A, Wiener H, Irvin MR, Zhi D, Limdi NA, Overton ET, Wassel CL, Divers J, Rotter JI, Post WS, Shrestha S (2017). Admixture mapping of subclinical atherosclerosis and subsequent clinical events among African Americans in 2 large cohort studies. Circ Cardiovasc Genet.

[32] Liu Y, Ao X, Ding W, Ponnusamy M, Wu W, Hao X, Yu W, Wang Y, Li P, Wang J (2018). Critical role of FOXO3a in carcinogenesis. Mol Cancer.

[33] Shiota M, Song Y, Yokomizo A, Kiyoshima K, Tada Y, Uchino H, Uchiumi T, Inokuchi J, Oda Y, Kuroiwa K (2010). Foxo3a suppression of urothelial cancer invasiveness through Twist1, Y-box-binding protein 1, and E-cadherin regulation. Clin Cancer Res.

[34] Shukla S, Bhaskaran N, Maclennan GT, Gupta S (2013). Deregulation of FoxO3a accelerates prostate cancer progression in TRAMP mice. Prostate.

[35] Tenbaum S, Ordóñez-Morán P, Puig I, Chicote I, Arqués O, Landolfi S, Fernández Y, Herance J, Gispert J, Mendizabal L (2012). β-Catenin confers resistance to PI3K and AKT inhibitors and subverts FOXO3a to promote metastasis in colon cancer. Nat Med.

[36] Sabine VS, Crozier C, Brookes CL, Drake C, Piper T, van de Velde CJ, Hasenburg A, Kieback DG, Markopoulos C, Dirix L (2014). Mutational analysis of PI3K/AKT signaling pathway in tamoxifen exemestane adjuvant multinational pathology study. J Clin Oncol.

[37] Lee YR, Chen M, Pandolfi PP (2018). The functions and regulation of the PTEN tumour suppressor: new modes and prospects. Nat Rev Mol Cell Biol.

[38] Li J, Yen C, Liaw D, Podsypanina K, Bose S, Wang SI, Puc J, Miliaresis C, Rodgers L, McCombie R (1997). PTEN, a putative protein tyrosine phosphatase gene mutated in human brain, breast, and prostate cancer. Science.

[39] Sansal I, Sellers WR (2004). The biology and clinical relevance of the PTEN tumor suppressor pathway. J Clin Oncol.

[40] Kun E, Tsang YTM, Ng CW, Gershenson DM, Wong KK (2021). MEK inhibitor resistance mechanisms and recent developments in combination trials. Cancer Treat Rev.

[41] Samatar AA, Poulikakos PI (2014). Targeting RAS-ERK signalling in cancer: promises and challenges. Nat Rev Drug Discov.

[42] Yaeger R, Corcoran RB (2019). Targeting alterations in the RAF-MEK pathway. Cancer Discov.

