# Supplementary material for: PIK3CA mutations-mediated downregulation of circLHFPL2 inhibits colorectal cancer progression via upregulating PTEN
Source: Mol Cancer. 2022 May 26;21:118. doi: 10.1186/s12943-022-01531-x (PMC9134670; doi:10.1186/s12943-022-01531-x)
Supplement: Supplementary file 1 — Additional file 1: Table S1. Primers and DNA/RNA srquence used in this study. [file 12943_2022_1531_MOESM1_ESM.docx]

| Table.S1 Primers and DNA/RNA srquence used in this study | |
| --- | --- |
| Item | Sequence（5‘-3’） |
| circLHFPL2 primer F (For qRT-PCR) | CCTTGGTGTCCGTCTTCAC |
| circLHFPL2 R (For qRT-PCR) | GGGAAGGAAGTCGCAGCT |
| circLHFPL2 divergent primer F (For PCR) | CCTGGCTGTGGGAATCTTTA |
| circLHFPL2 divergent primer R (For PCR) | AGTCGGTGGGGAAGGAGG |
| circLHFPL2 convergent primers F (For PCR) | TCCTGGCTGTGGGAATCTTT |
| circLHFPL2 convergent primers R (For PCR) | AATTCCTTGCAACAGCCCAC |
| GADPH divergent primer F (For PCR) | CCAGAACATCATCCCTGCCT |
| GADPH divergent primer R (For PCR) | TCCACCACTGACACGTTGG |
| GADPH convergent primers F (For PCR) | AGAAGACTGTGGATGGCCC |
| GADPH convergent primers R (For PCR) | CCAGTGAGCTTCCCGTTCA |
| GAPDH primer F (For qRT-PCR) | GGAGCGAGATCCCTCCAAAAT |
| GAPDH primer R (For qRT-PCR) | GGCTGTTGTCATACTTCTCATGG |
| hsa-miR-1322 primer F (For qRT-PCR) | GATGATGCTGCTGATGCTG |
| hsa-miR-1322 primer R (For qRT-PCR) | CGTATCCAGTGCGTGTCGTG |
| hsa-miR-556-5p primer F (For qRT-PCR) | GATGAGCTCATTGTAATATGAG |
| hsa-miR-556-5p primer R (For qRT-PCR) | CGTATCCAGTGCGTGTCGTG |
| U6 primer F (For qRT-PCR) | CTCGCTTCGGCAGCACA |
| U6 primer R (For qRT-PCR) | AACGCTTCACGAATTTGCGT |
| miR-1322 mimics | GAUGAUGCUGCUGAUGCUG |
| miR-556-5p mimics | GAUGAGCUCAUUGUAAUAUGAG |
| si-circLHFPL2 #1 | AAGGAATTGCAGGTGAGGCCG |
| si-circLHFPL2 #2 | GCAAGGAATTGCAGGTGAGGC |
| siPTEN #1 | CAGTAGAAATTGTCCTACATGTGCT |
| siPTEN #2 | CGGCAGCATCAAATGTTTCAGCTTT |
