# Supplementary material for: PIK3CA mutations-mediated downregulation of circLHFPL2 inhibits colorectal cancer progression via upregulating PTEN
Source: Mol Cancer. 2022 May 26;21:118. doi: 10.1186/s12943-022-01531-x (PMC9134670; doi:10.1186/s12943-022-01531-x)
Supplement: Supplementary file 2 — Additional file 2: Table S2. Antibody information. [file 12943_2022_1531_MOESM2_ESM.docx]

| Table. S2 Antibody information | | | | | |
| --- | --- | --- | --- | --- | --- |
| Antibody | Brand | Cat. No | Dilution Rate | MW (kDa) | Species |
| PTEN | CST | 9188 | 1:1000 | 54 | Rabbit IgG |
| Bcl-2 | CST | 4223 | 1:1000 | 26 | Rabbit IgG |
| Bax | CST | 2774 | 1:1000 | 20 | Rabbit IgG |
| cleaved-PARP | CST | 5625 | 1:1000 | 89 | Rabbit IgG |
| p-Foxo3a | CST | 9466 | 1:500 | 97 | Rabbit IgG |
| p-AKT（473） | CST | 4060 | 1:500 | 60 | Rabbit IgG |
| GAPDH | CST | 5174 | 1:1000 | 37 | Rabbit IgG |
