# Supplementary material for: PIK3CA mutations-mediated downregulation of circLHFPL2 inhibits colorectal cancer progression via upregulating PTEN
Source: Mol Cancer. 2022 May 26;21:118. doi: 10.1186/s12943-022-01531-x (PMC9134670; doi:10.1186/s12943-022-01531-x)
Supplement: Supplementary file 3 — Additional file 3: Table S3. The list of predicted miRNAs. [file 12943_2022_1531_MOESM3_ESM.docx]

Table S3 The list of predicted miRNAs

| miRNA | Aligment |
| --- | --- |
| hsa-miR-585-3p | 5' cccuggGCAUCUACGCCCg 3'          :\|\|  \|\|\|\|\|\|\|  3' aucguaUGUCUAUGCGGGu 5' |
| hsa-miR-510 | 5'AUGCUCUGGACCUUGCUGAGUAU 3'                \|\|\|\|\|\|   3'CACUAACGGUGAGAGGACUCAU 5' |
| hsa-miR-198 | 5'CCUGUCGCUCGAUGCUCUGGACC 3'                 \|\|\|\|\|\|\|  3' CUUGGAUAGAGGGGAGACCUGG 5' |
| hsa-miR-1322 | 5'CCAUGUGUGUACAGAGCAUCAUG 3'                 \|\|\|\|\|\|\|  3'    GUCGUAGUCGUCGUAGUAG 5' |
| hsa-miR-1252 | 5'GACUGCAGCUGCGACUUCCUUCC 3'                  \|\|\|\|\|\|\|  3' AUUUACUUAAGUUAAAGGAAGA 5' |
| hsa-miR-1279 | 5' CUUCGCCCGGAACAU—CAAUAUGU 3'            \|\|\|    \|\|\|\|\|\|\|  3'       UCUUUCUUCGUUAUACU 5' |
| hsa-miR-622 | 5' UUGCCUUCAUGAGUGCAGACUGG 3'                  \|\|\|\|\|\|\|  3'   CGAGGUUGGAGUCGUCUGACA 5' |
| hsa-miR-545 | 5' UCGAUGCUCUGGACCUUGCUGAG 3'                    \|\|\|\|\|\|   3' CGUGUGUUAUUUACAAACGACU 5' |
| hsa-miR-556-5p | 5' UGGUGGCUUUUGCCGAGCUCAUU 3'                 \|\|\|\|\|\|\|  3'  GAGUAUAAUGUUACUCGAGUAG 5' |
| hsa-miR-433 | 5' AUGUGUGUACAGAGC—AUCAUGAA 3'             \|\|\|\|  \|\|\|\|\|\|\|  3'    UGUGGCUCCUCGGGUAGUACUA 5' |
