# Supplementary figures and images for: PIK3CA mutations-mediated downregulation of circLHFPL2 inhibits colorectal cancer progression via upregulating PTEN
Source: Mol Cancer. 2022 May 26;21:118. doi: 10.1186/s12943-022-01531-x (PMC9134670; doi:10.1186/s12943-022-01531-x)

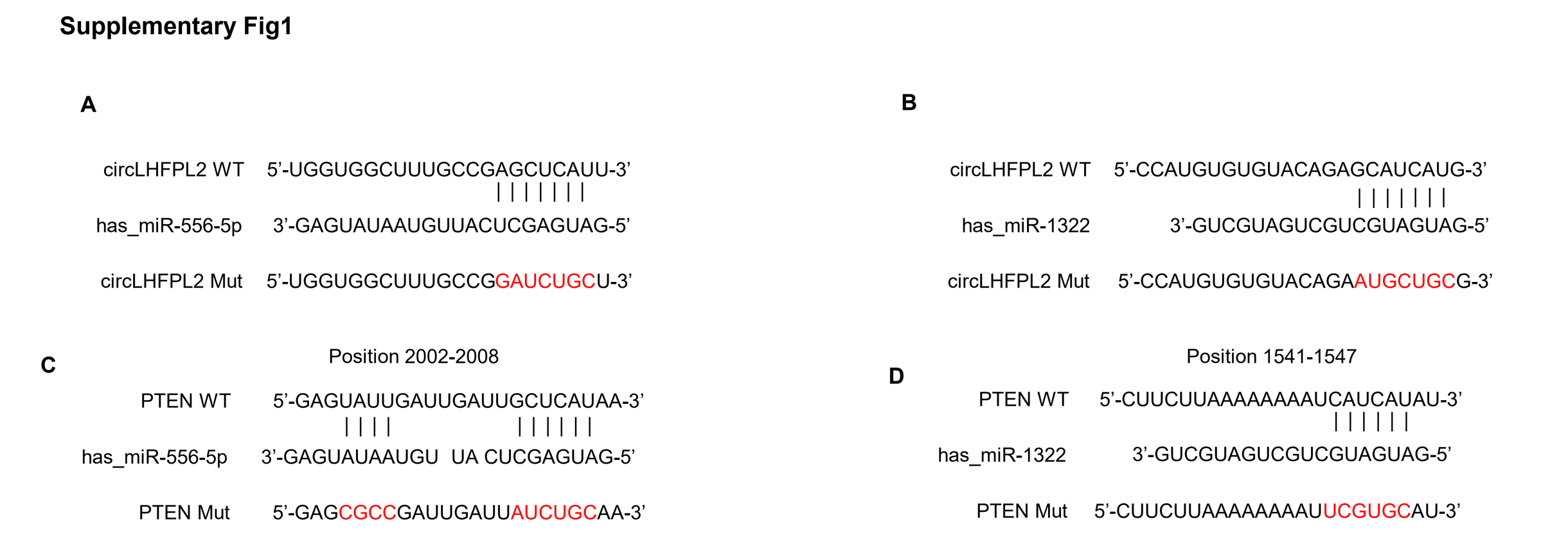

Supplement: Supplementary file 4 — Additional file 4: Supplementary Fig. 1. Predicted binding sites. (A) Predicted binding site between circLHFPL2 and miR-556-5p. (B) Predicted binding site between circLHFPL2 and miR-1322. (C) Predicted binding site between PTEN and miR-556-5p. (D) Predicted binding site between PTEN and miR-1322. [file 12943_2022_1531_MOESM4_ESM.tif]

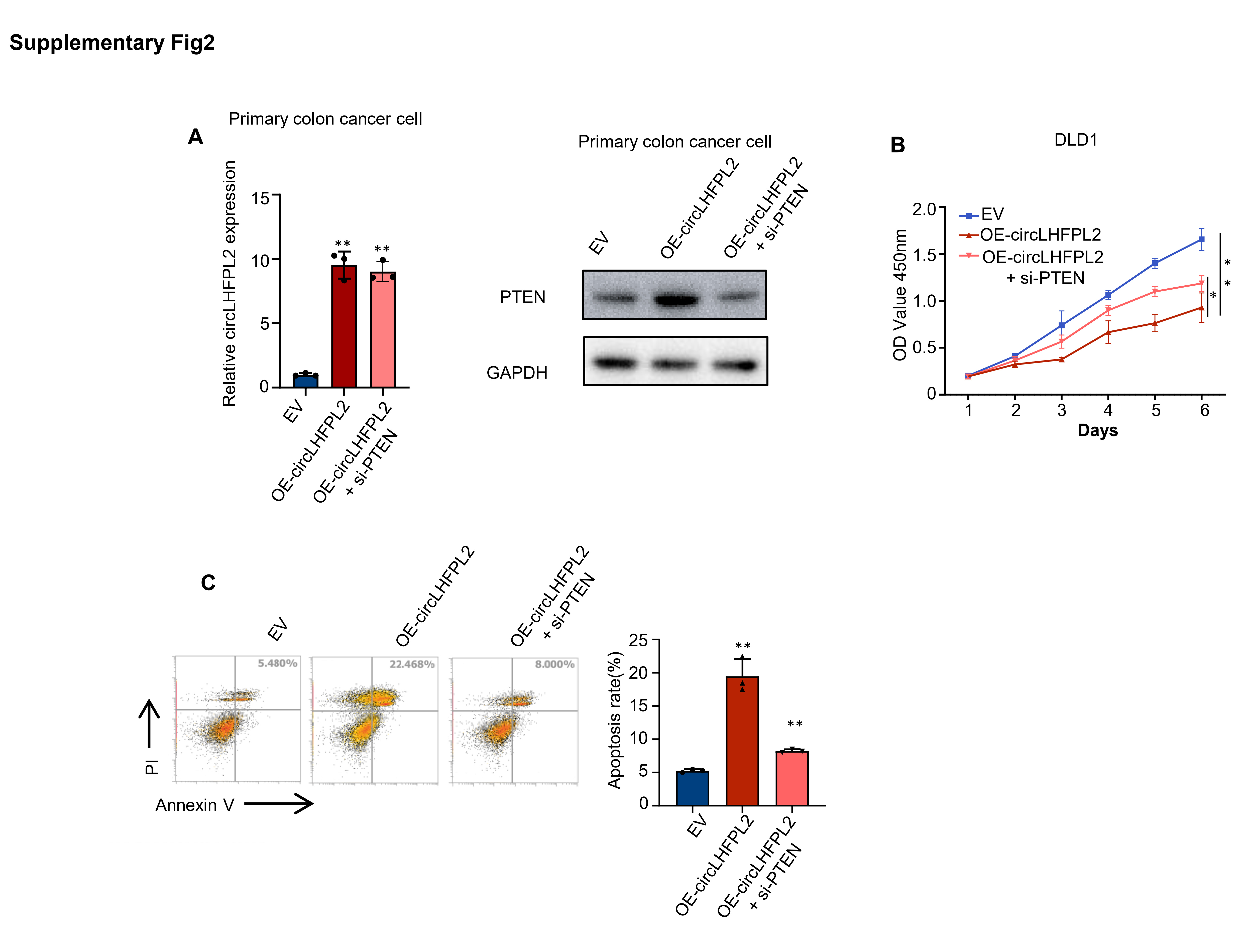

Supplement: Supplementary file 5 — Additional file 5: Supplementary Fig. 2. Overexpression of circLHFPL2 inhibits primary colon cancer cell proliferation and promotes apoptosis by regulating PTEN. (A) circLHFPL2 expression detected by qRT-PCR in cells transfected with circLHFPL2 overexpression plasmid or co-transfected with si-PTEN (left panel). PTEN expression was detected by Western blot in cells transfected with circLHFPL2 overexpression plasmid or co-transfected with si-PTEN (right panel). (B) CCK8 assays examined the proliferation of cells transfected with circLHFPL2 overexpression plasmid or co-transfected with si-PTEN. (C) Apoptosis assays examined the apoptosis rate of cells transfected with circLHFPL2 overexpression plasmid or co-transfected with si-PTEN. Data are presented as mean ± SEM; n ≥ 3. *p < 0.05; **p < 0.01. [file 12943_2022_1531_MOESM5_ESM.tif]

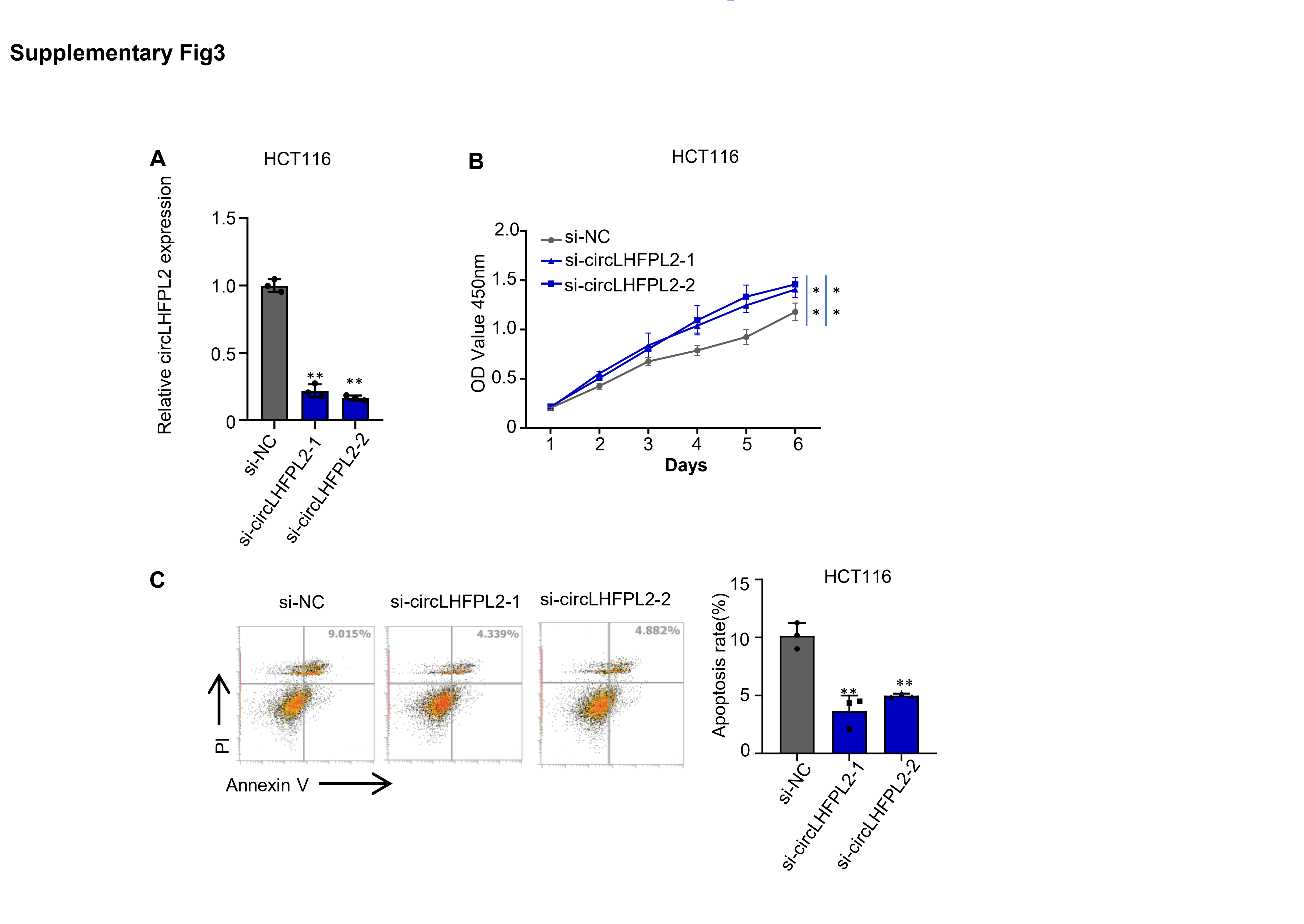

Supplement: Supplementary file 6 — Additional file 6: Supplementary Fig. 3. Silencing circLHDPL2 promotes the viability of HCT116 cells. (A) circLHFPL2 expression detected by qRT-PCR after siRNA which targets circLHFPL2 in HCT116 cells. (B) CCK8 assay was used to examine the proliferation of HCT116 cells transfected with siRNA which targets circLHFP2L. (C) Apoptosis assay was used to examine the apoptosis of HCT116 cells transfected with siRNA which targets circLHFP2L. Data are presented as mean ± SEM; n ≥ 3. *p < 0.05; **p < 0.01. [file 12943_2022_1531_MOESM6_ESM.tif]

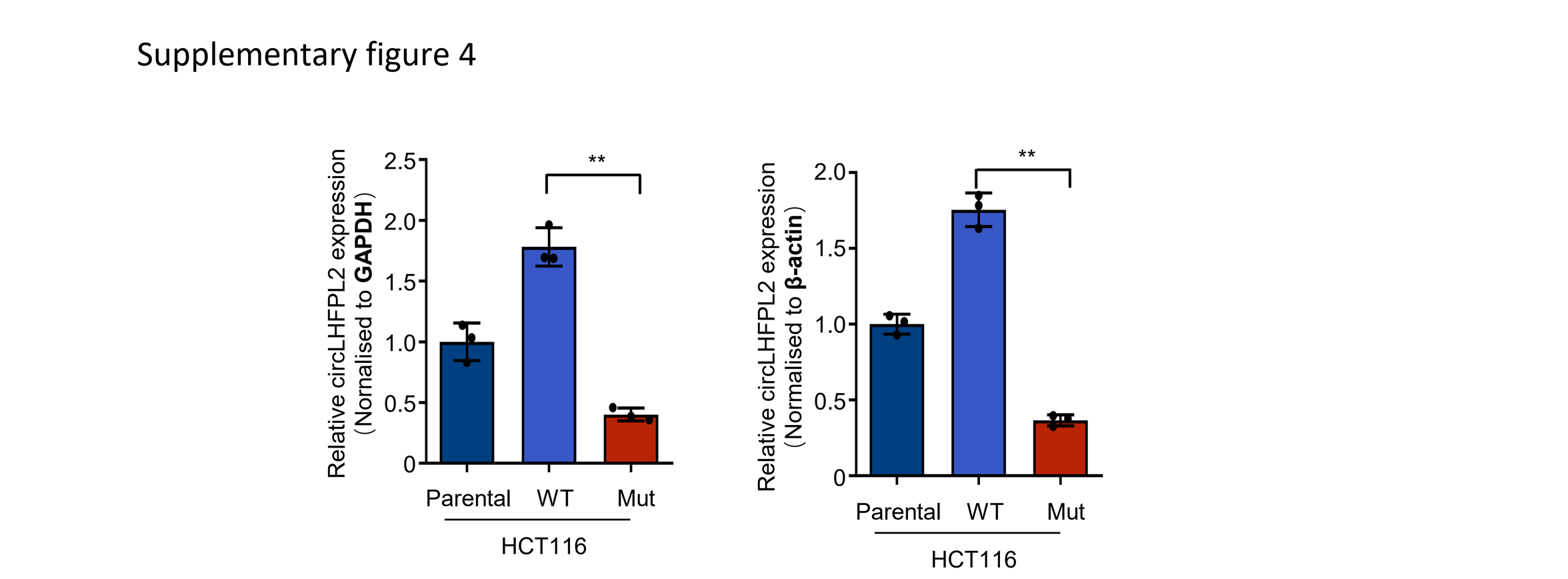

Supplement: Supplementary file 7 — Additional file 7: Supplementary Fig. 4. The expression of circLHFPL2 in HCT116 mutant and WT clones normalized to GAPDH and β-actin. [file 12943_2022_1531_MOESM7_ESM.tif]

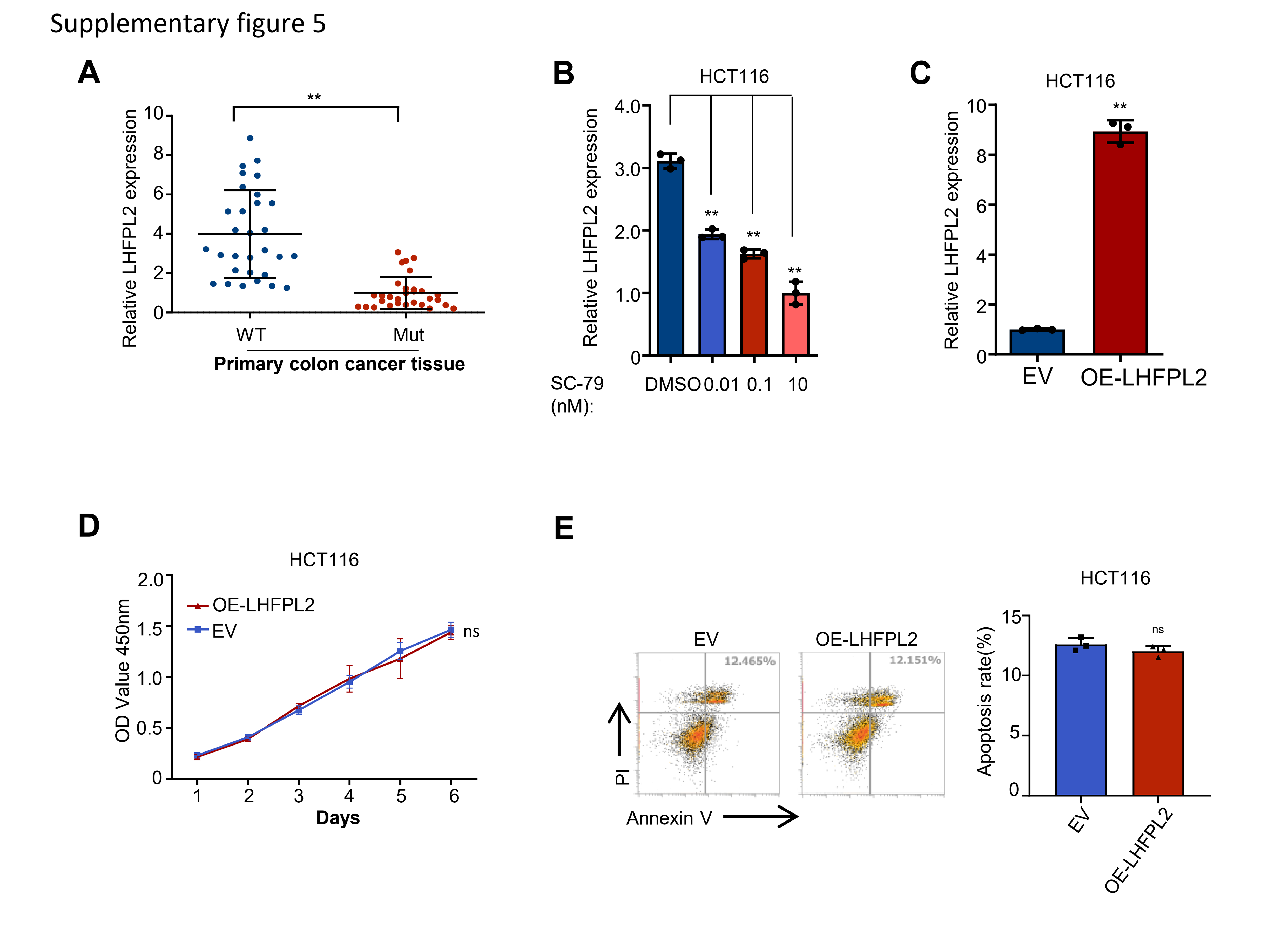

Supplement: Supplementary file 8 — Additional file 8: Supplementary Fig. 5. Overexpression of linear LHFPL2 has no effect on HCT116 cell viability. (A) Linear LHFPL2 expression in 30 PIK3CA-mutant and 30 WT CRC tissues. (B) Linear LHFP2 was downregulated upon SC-79 treatment. (C) qRT-PCR detected the expression of linear LHFPL2 expression in HCT116 cells transfected with LHFPL2 overexpression plasmids. (D) CCK8 assay examined the proliferation of HCT116 cells transfected with LHFPL2 overexpression plasmids. (E) Apoptosis assay examined the apoptotic rate of HCT116 cells transfected with LHFPL2 overexpression plasmids. Data are presented as mean ± SEM; n ≥ 3. *p < 0.05; **p < 0.01. [file 12943_2022_1531_MOESM8_ESM.tif]

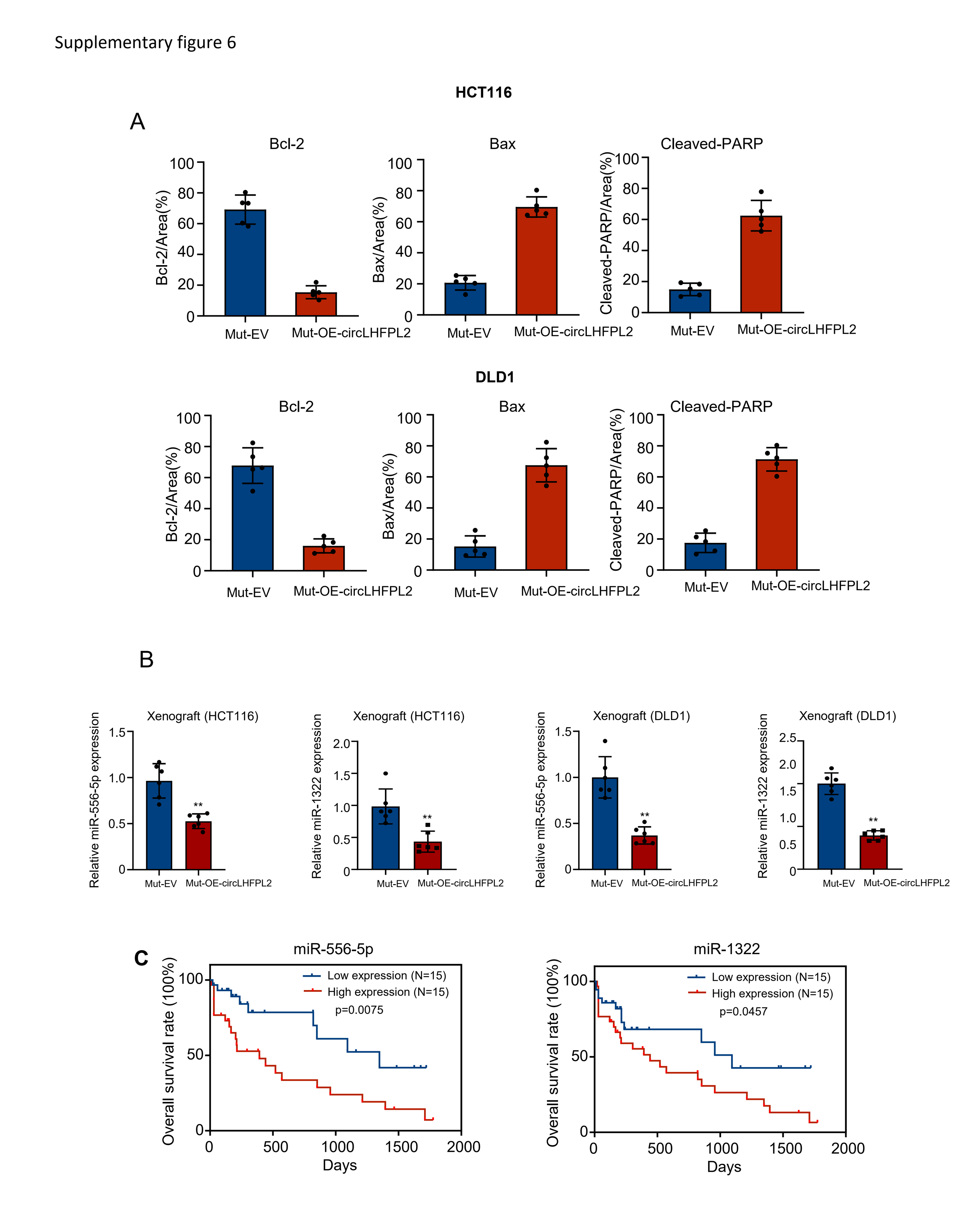

Supplement: Supplementary file 9 — Additional file 9: Supplementary Fig. 6. (A) Relative quantification of Bcl-2, Bax, cleaved PARP for Fig. 2B. (B) miR-556-5p and miR-1322 expression in xenografted tumors. (C) Survival analysis in patients with different expression pattern of miR-556-5p (left) and miR-1322 (right) with Kaplan-Meier method. Data are presented as mean ± SEM; n ≥ 3. *p < 0.05; **p < 0.01. [file 12943_2022_1531_MOESM9_ESM.tif]

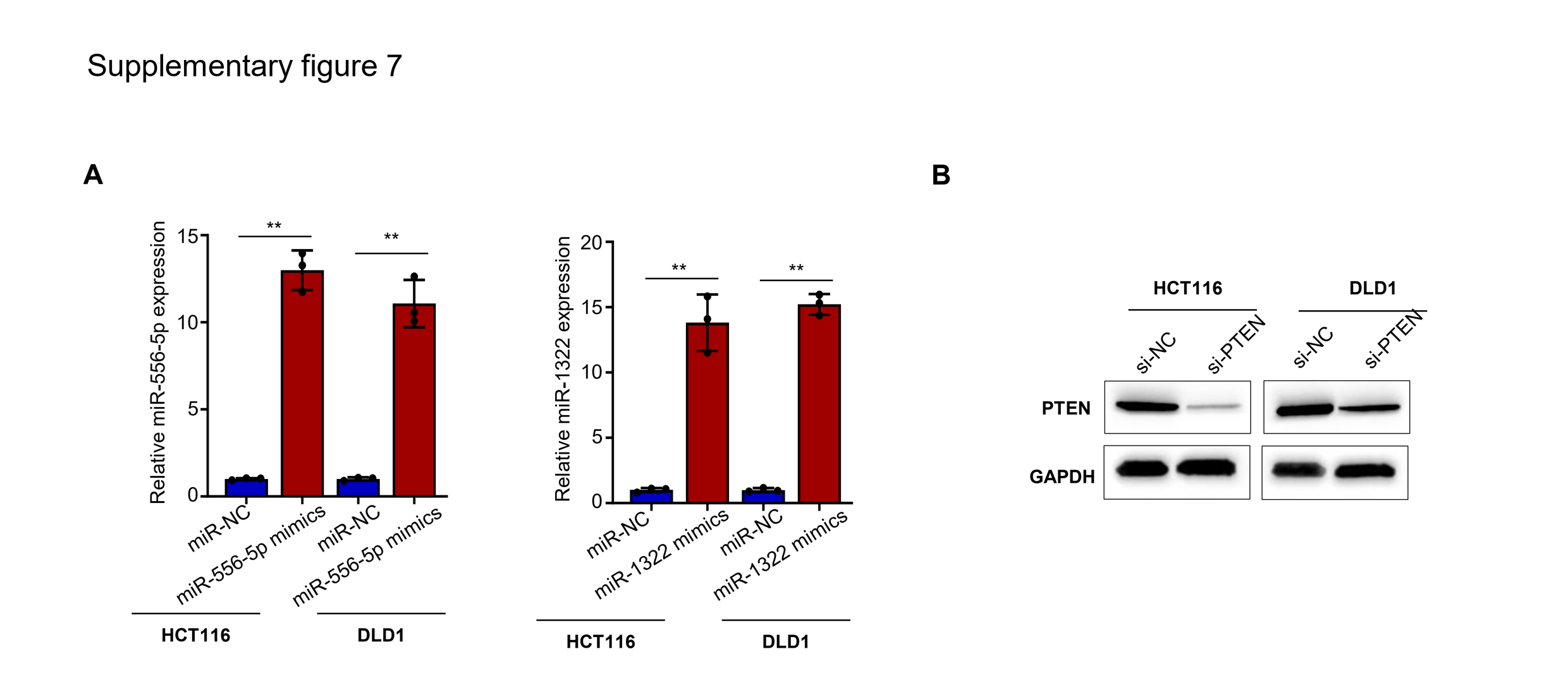

Supplement: Supplementary file 10 — Additional file 10: Supplementary Fig. 7. The transfection efficiency of miR-556-5p mimics (A, left), miR-1322 mimics (A, right) and siRNA which targets PTEN (B). Data are presented as mean ± SEM; n ≥ 3. *p < 0.05; **p < 0.01. [file 12943_2022_1531_MOESM10_ESM.tif]

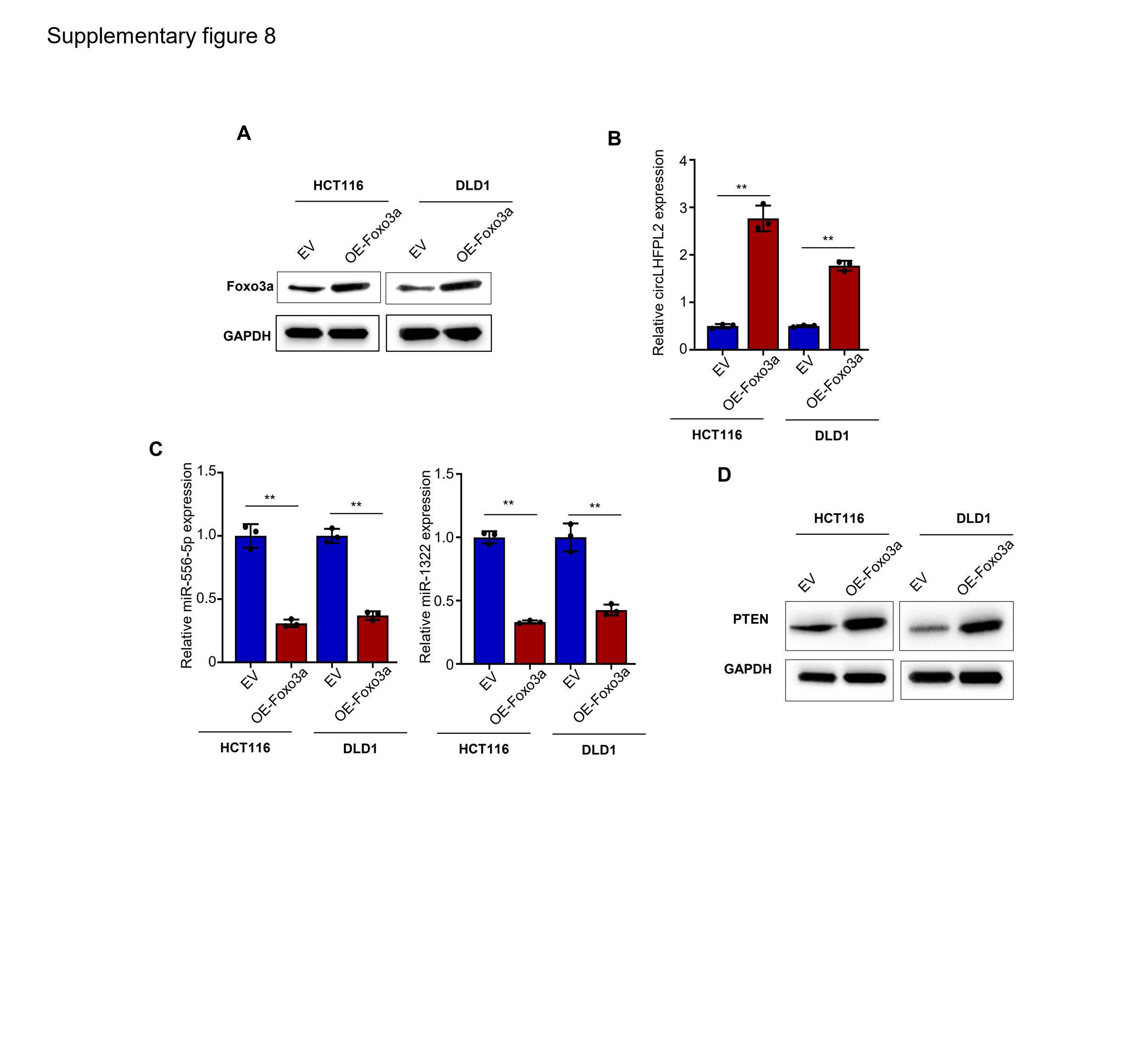

Supplement: Supplementary file 11 — Additional file 11: Supplementary Fig. 8. The effect of overexpression of FOXO3a on circLHFPL2, miR-556-5p, miR-1322 and PTEN expression. (A) Western blot detected the FOXO3a expression in HCT116 and DLD1 cells transfected with FOXO3a overexpression plasmids. (B) qRT-PCR detected the circLHFPL2 expression in HCT116 and DLD1 cells transfected with FOXO3a overexpression plasmids. (C) qRT-PCR detected the miR-556-5p and miR-1322 expression in HCT116 and DLD1 cells transfected with FOXO3a overexpression plasmids. (D) Western blot detected the PTEN expression in HCT116 and DLD1 cells transfected with FOXO3a overexpression plasmids. [file 12943_2022_1531_MOESM11_ESM.tif]
